# Lineage tracing reveals the origins and dynamics of macrophages in lung injury and repair

**DOI:** 10.1038/s41421-025-00859-0

**Published:** 2026-01-14

**Authors:** Hengwei Jin, Jialing Mou, Huan Zhu, Kuo Liu, Mingjun Zhang, Zhenqian Zhang, Stefan Pflanz, Karim EI Kasmi, Zhaoyuan Liu, Florent Ginhoux, Kathy O. Lui, Bin Zhou

**Affiliations:** 1 CAS CEMCS-CUHK Joint Laboratories for Cardiovascular Sciences, New Cornerstone Investigator Laboratory, Key Laboratory of Multi-Cell Systems, Shanghai Institute of Biochemistry and Cell Biology, Center for Excellence in Molecular Cell Science, Chinese Academy of Sciences; University of Chinese Academy of Sciences, Shanghai, China; 2 Key Laboratory of Systems Health Science of Zhejiang Province, School of Life Science, Hangzhou Institute for Advanced Study, University of Chinese Academy of Sciences, Hangzhou, Zhejiang, China; 3 Boehringer Ingelheim Pharma GmbH & Co KG, Biberach an der Riss, Germany; 4 Department of Immunology and Microbiology, Shanghai Institute of Immunology, Shanghai Jiao Tong University School of Medicine, Shanghai, China; 5 Singapore Immunology Network, Agency for Science, Technology and Research, Singapore, Singapore; 6 CAS CEMCS-CUHK Joint Laboratories for Cardiovascular Sciences, Department of Chemical Pathology, and Li Ka Shing Institute of Health Science, Prince of Wales Hospital, The Chinese University of Hong Kong, Hong Kong, China; 7 School of Life Science and Technology, ShanghaiTech University, Shanghai, China

**Keywords:** Immunology, Cell biology

## Abstract

Macrophages play a vital role in tissue repair and regeneration following injury. However, the cell fate, dynamic responses, and functions of macrophages from various origins during lung injury and repair are not fully understood. Here, we used genetic lineage tracing and scRNA-seq approaches to explore the temporal and spatial roles of tissue-resident and infiltrating macrophages during pulmonary fibrosis. We observed a sharp reduction in tissue-resident macrophages during the early inflammatory phase, with their numbers stabilizing during recovery. Monocytes contributed substantially to the macrophage population during the fibrotic phase, initially differentiating into interstitial macrophages and later transitioning into alveolar macrophages through a transient state. Genetic ablation of monocytes led to a reduction in the number of infiltrating macrophages and alleviated pulmonary fibrosis. Mechanistically, Notch signaling was negatively correlated with Wnt/β-catenin signaling in the regulation of monocyte recruitment and pulmonary fibrosis. Our study reveals the dynamic contributions and functions of macrophages from various sources in lung injury and regeneration.

## Introduction

In recent years, pulmonary diseases have emerged as a significant and growing cause of disability and mortality among adults^1^. Acute lung injury can lead to the development of several respiratory conditions, including idiopathic pulmonary fibrosis, chronic obstructive pulmonary disease, asthma, and alveolar sarcoma^2^. These conditions are believed to result from defective pulmonary epithelium development, basement membrane denudation, inflammatory cell influx, and macrophage and neutrophil activation^3,4^. Macrophages play a critical role in the innate immune system, with their widespread distribution across different organs. They are essential for maintaining tissue homeostasis and immune surveillance, clearing cellular debris, and orchestrating inflammatory responses that protect against infection^5–8^.

At steady state, tissue macrophages in the brain, epidermis, lungs, and liver are generated during embryonic hematopoiesis, arising independently of bone marrow (BM) contributions, and persist into adulthood^9–12^. These tissue-resident macrophages (TRMs) are maintained and expanded through local proliferation rather than through recruitment from circulating monocytes^13,14^. In contrast, macrophages in the gut and dermis are replaced more rapidly by circulating monocytes^15^. Notably, the diverse functions of macrophages during tissue homeostasis and inflammation are highly dependent on their origin, ontogeny, and anatomical location.

Lung macrophages exhibit considerable heterogeneity and remarkable plasticity^2,16^. In the lungs, two major subpopulations of macrophages coexist: alveolar macrophages (AMs) and interstitial macrophages (IMs)^17^. These populations are distinguished by their specific location, characteristics, and functions. AMs are located primarily in the airways and are characterized by the expression of CD11c and SiglecF, but lack CD11b expression. In contrast, IMs are predominantly found in the lung parenchyma and express high levels of CD11b^16,18^. In a steady state, the number of AMs is significantly greater than that of IMs in the lungs^19^. Resident AMs are established before birth, are largely derived from embryonic hematopoiesis in the yolk sac and/or fetal liver, and are maintained through local proliferation. In contrast, IMs are gradually replaced by blood monocytes^20^.

In response to inflammatory stimuli, in addition to TRMs, monocytes are recruited to the site of injury, where they differentiate into macrophages with distinct polarization states, namely, M1-like and M2-like macrophages. In these monocyte-derived macrophages (MDMs), M1 macrophages primarily mediate proinflammatory responses and produce IL1β, IL6, and TNFα. In contrast, M2 macrophages are predominantly associated with inflammation resolution and produce anti-inflammatory factors such as Arg1, IL10, and Chil3^21,22^.

The mechanisms by which TRMs and MDMs are altered in response to injury during repair, as well as the specific functions of these distinct cell populations, have not been thoroughly investigated. Previous studies have relied primarily on fluorescence-activated cell sorting (FACS) using a series of cell-specific markers or on bone marrow cell transplantation models^13,23–25^. However, the heterogeneity of macrophages remains unexplored, largely because of the absence of genetic tools that can specifically target macrophages in different organs. Bleomycin is a well-characterized and widely used model for inducing progressive pulmonary inflammation and subsequent fibrosis, which aids in understanding the pathogenesis and pathophysiology of acute lung injury and acute respiratory distress syndrome^3,26–28^. In this study, we aimed to characterize the dynamic responses and functions of macrophages of various origins, both at steady state and following bleomycin-induced lung injury. Additionally, we investigated the molecular mechanisms contributing to bleomycin-induced lung injury during both the inflammatory phase and the fibrotic phase. This study not only introduces specific genetic tools to enhance our understanding of the dynamic changes in macrophages from different origins in response to injury but also reveals their functions and potential molecular regulatory mechanisms targeting lung repair and regeneration.

## Results

### Generation of a *CD68-rtTA* mouse line for lineage tracing of TRMs

To overcome the limitations of previous approaches in distinguishing between TRMs and MDMs after lung injury^8,29,30^, we generated a *CD68-rtTA* mouse line for efficient lineage tracing of TRMs. Earlier studies relied on CD45.1/CD45.2 bone marrow chimeras, predominantly used in cell transplantation^13,23^, which cannot specifically target TRMs during lung homeostasis. To address this, we initially employed the *CX3CR1-CreER* system^31,32^ to label TRMs through tamoxifen (Tam) induction at the embryonic and neonatal stages. This method resulted in low labeling efficiency and poor survival rates, particularly when Tam was administered during embryogenesis (Supplementary Fig. S1a–f). Thus, we sought to improve TRM labeling by combining the tetracycline-on (Tet-On) and Cre-*loxP* systems to increase labeling efficiency in adult mice.

**Fig. 1:**
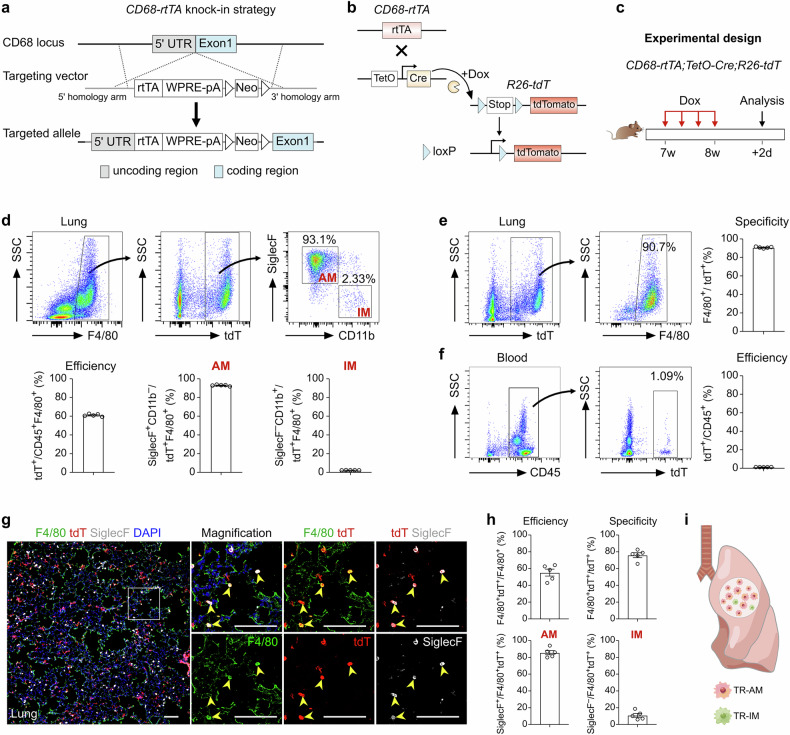
Generation and characterization of the *CD68-rtTA* mouse line. **a** Schematic diagram showing the knock-in strategy of the *CD68-rtTA* line by homologous recombination. **b** Genetic lineage tracing strategy by Cre-*loxP* recombination in CD68^+^ cells after doxycycline (Dox) treatment. **c** Schematic showing the experimental design. **d** Flow cytometric and quantification analyses of the percentage of tdT^+^ cells among CD45^+^F4/80^+^ macrophages (efficiency) and the percentage of SiglecF^+^ alveolar macrophages (AMs, left) and CD11b^+^ interstitial macrophages (IMs, right) among tdT^+^ macrophages from the lungs. Data are presented as the mean ± SEM; *n* = 5 mice per group. **e** Flow cytometric and quantification analyses of the percentage of tdT^+^ cells expressing F4/80 (specificity) in the lungs. Data are presented as the mean ± SEM; *n* = 5 mice per group. **f** Flow cytometric and quantification analyses of the percentage of CD45^+^ monocytes expressing tdT from blood. Data are presented as the mean ± SEM; *n* = 5 mice per group. **g** Immunostaining for F4/80, tdT, and SiglecF in lung tissue sections. The boxed region is magnified. Yellow arrowheads indicate labeled macrophages. **h** Quantitative analysis of the percentage of tdT^+^ cells among F4/80^+^ macrophages (efficiency), the percentage of F4/80^+^ cells among tdT^+^ cells (specificity), and the percentage of AMs and IMs among labeled tdT^+^ macrophages from images of immunostained lung sections. Data are presented as the mean ± SEM; *n* = 5 mice per group. **i** Cartoon image showing the effective labeling of tissue-resident macrophages (TRMs) in *CD68-rtTA;TetO-Cre;R26-tdT* mice. Scale bars, 100 µm. Each image is representative of five individual samples.

Analysis of single-cell RNA sequencing (scRNA-seq) data from lung tissue revealed high expression of CD68 across macrophage populations, with the majority of AMs exhibiting strong CD68 expression (Supplementary Fig. S2a). Based on this expression pattern, we generated *CD68-rtTA* mice, in which rtTA expression is controlled by the CD68 promoter in macrophages (Fig. 1a). In this system, *TetO-Cre* induces Cre recombinase expression under the control of a minimal promoter containing the tet operator (tetO). Upon doxycycline (Dox) administration, rtTA binds to *TetO-Cre*, driving Cre expression and subsequently activating a reporter gene in CD68^+^ macrophages (Fig. 1b). In the absence of Dox, rtTA does not bind to the tetO promoter, preventing the continuous or uncontrolled activation of Cre.

To validate this system, we crossed *CD68-rtTA*, *TetO-Cre*, and *R26-tdT* mice to generate a triple-positive mouse line and analyzed macrophage labeling after 1 week of Dox treatment in adult mice (Fig. 1c). FACS analysis revealed that more than 60% of lung macrophages expressing F4/80 were labeled with tdT, while nearly all CD45^+^ immune cells from blood remained tdT-negative (Fig. 1d, f). Furthermore, 90.75 ± 0.35% of the tdT^+^ cells in the lungs expressed F4/80, confirming the high specificity of the *CD68-rtTA;TetO-Cre;R26-tdT* system for labeling macrophages (Fig. 1e). Among the labeled tdT^+^ macrophages, the majority were AMs (92.94 ± 0.33%), while IMs were minimally labeled (2.31 ± 0.05%, Fig. 1d). Notably, more than 70% of all AMs were tdT-positive, compared with only approximately 7.58% of IMs, which is consistent with the known predominance of AMs within homeostatic TRM populations (Supplementary Fig. S2b). Immunostaining of lung sections for tdT, F4/80, and SiglecF further demonstrated greater labeling efficiency and specificity in *CD68-rtTA;TetO-Cre;R26-tdT* mice than in *CX*_*3*_*CR1-CreER;R26-tdT* mice (Fig. 1g, h). In addition, by employing CD64 and MerTK as specific markers to accurately identify TRMs in the lung, FACS analysis demonstrated that more than 40% of the TRMs were tdT^+^, with the vast majority being AMs (96.07 ± 1.54%), whereas IMs showed minimal labeling (3.79 ± 1.12%). Furthermore, 95.01 ± 2.02% of all the tdT^+^ cells coexpressed CD64 and MerTK, confirming the high specificity of this system for macrophage labeling (Supplementary Fig. S2c, d). Immunostaining further confirmed the specificity of the system, demonstrating that lung tdT^+^ cells coexpressed CD68 and MerTK and were positive for the AM marker SiglecF, while no detectable tdT or CD68 signal was detected in blood Ly6C^+^ monocytes after Dox treatment (Supplementary Fig. S2e). Importantly, negligible tdT signals were detected in *CD68-rtTA;TetO-Cre;R26-tdT* mice without Dox treatment (Supplementary Fig. S3). Whole-mount fluorescence and immunofluorescence imaging further confirmed that sporadic tdT^+^ cells were not F4/80^+^ macrophages (Supplementary Fig. S3b, c). Additionally, FACS analysis revealed that fewer than 0.01% of F4/80^+^ macrophages and Ly6C^+^ monocytes expressed tdT in the absence of Dox (Supplementary Fig. S3d). In summary, the *CD68-rtTA;TetO-Cre;R26-tdT* mouse line offers a highly specific and efficient tool for the genetic targeting and lineage tracing of endogenous TRMs in the lungs (Fig. 1i). This model provides a valuable resource for studying the roles of TRMs in pulmonary homeostasis and disease.

### TRMs decrease significantly after injury and are maintained at normal levels during recovery

**Fig. 2:**
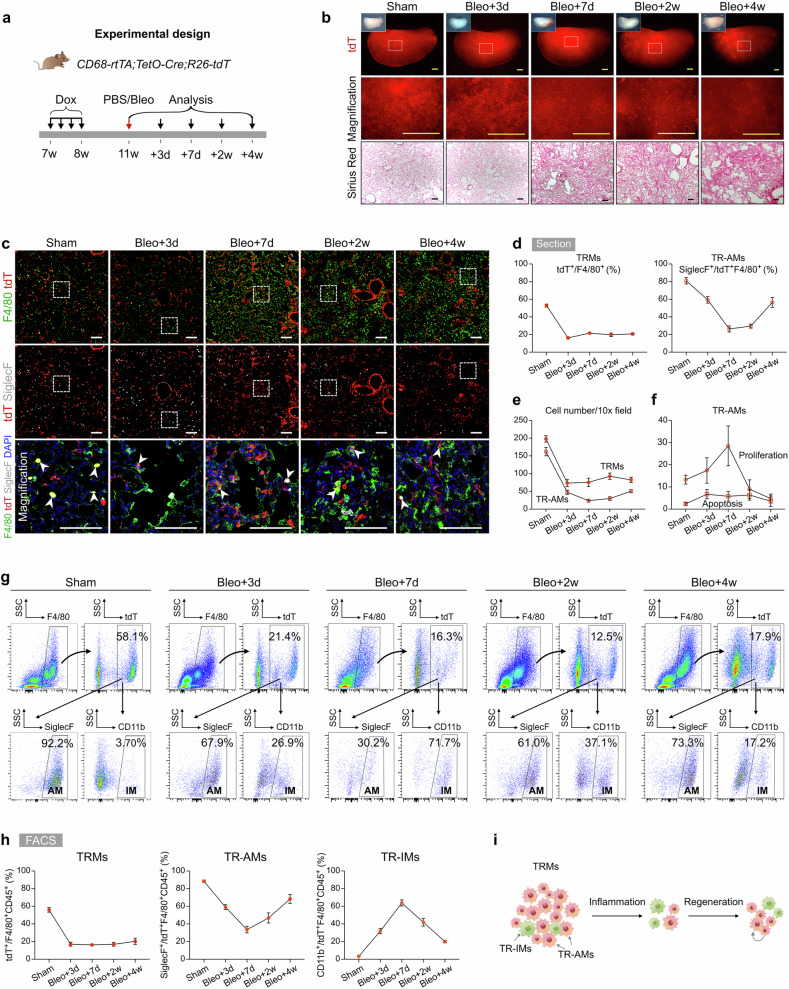
Lineage tracing of TRMs reveals significantly reduced TRMs after bleomycin-induced injury. **a** Schematic diagram showing the experimental design. Dox, doxycycline. Bleo, bleomycin. **b** Whole-mount bright field, epifluorescence, and Sirius red images of lungs after bleomycin treatment. The boxed region is magnified. **c** Immunostaining for tdT, F4/80, and SiglecF in lung tissue sections. The boxed region is magnified. White arrowheads indicate TRMs. **d** Quantitative analysis of the percentage of F4/80^+^ macrophages expressing tdT (left) and the percentage of SiglecF^+^ alveolar macrophages among labeled tdT^+^ macrophages (right) in immunostained lung sections. Data are presented as the mean ± SEM; *n* = 5 mice per group. **e** Quantification of the number of TRMs (tdT^+^F4/80^+^) and TR-AMs (tdT^+^SiglecF^+^) in each 10× field from immunostained lung sections. Data are presented as the mean ± SEM; *n* = 5 mice per group. **f** Quantification of the percentage of Ki67^+^ (proliferative) and γ-H2AX^+^ (apoptotic) cells among labeled TR-AMs (tdT^+^SiglecF^+^) in each 10× field from immunostained lung sections. Data are presented as the mean ± SEM; *n* = 5 mice per group. **g**, **h** Flow cytometric analysis and quantification of the percentage of tdT^+^ cells among CD45^+^F4/80^+^ macrophages (TRMs), the percentage of SiglecF^+^ alveolar macrophages among tdT^+^ macrophages (TR-AMs), and the percentage of CD11b^+^ interstitial macrophages among tdT^+^ macrophages (TR-IMs) from the lungs. Data are presented as the mean ± SEM; *n* = 5 mice per group. **i** Cartoon image showing the dynamic changes in TRMs in the lungs after bleomycin-induced injury. TRMs tissue-resident macrophages, TR-AMs tissue-resident alveolar macrophages, TR-IMs tissue-resident interstitial macrophages. Scale bars, yellow, 1 mm; white and black, 100 µm. Each image is representative of five individual samples.

To investigate the role and dynamics of TRMs in lung injury and repair, we induced acute lung inflammation and fibrosis through bleomycin induction. In this model, the levels of proinflammatory cytokines rapidly increase during the first 3 days, peak at approximately day 7, and remain elevated until day 10, followed by the fibrotic phase of lung recovery^28^. To assess the behavior of TRMs during these stages, we treated *CD68-rtTA;TetO-Cre;R26-tdT* mice with Dox to label TRMs, followed by a 3-week Dox washout period before administering bleomycin. The lungs were then harvested at different time points: 3, 7, 14, and 28 days postinjury (Fig. 2a). Whole-mount fluorescence images of the lungs of *CD68-rtTA;TetO-Cre;R26-tdT* mice revealed a significant decrease in the number of tdT-labeled TRMs over time after injury (Fig. 2b). Sirius red staining of lung sections revealed a sharp increase in fibrosis starting on day 7 postinjury, with the severity of fibrosis worsening at days 14 and 28 compared with that of the sham control (Fig. 2b).

Next, we performed immunofluorescence staining of lung sections for tdT, F4/80, and SiglecF and found that the percentage of TRMs among total macrophages was significantly decreased by day 3 postinjury and remained relatively constant throughout the later stages of injury and recovery (Fig. 2c, d; Supplementary Fig. S4). Concurrently, we observed a marked increase in the number of tdT^–^F4/80^+^ macrophages from day 7 to day 28, suggesting infiltration of macrophages from other sources, particularly during the fibrotic phase (Fig. 2c; Supplementary Fig. S4). The percentage of TR-AMs among the tdT^+^ TRMs initially decreased on days 3 and 7 but then gradually increased to 56.44 ± 5.71% by day 28 (Fig. 2d). During the fibrotic phase, especially on day 28, there was a notable increase in the number of TR-AMs compared with that in the earlier inflammatory phase, whereas the overall number of TRMs remained stable (Fig. 2e). The proliferation of TR-AMs peaked on day 7 and returned to baseline levels by day 14, with minimal changes in apoptosis over time (Fig. 2f; Supplementary Fig. S5), suggesting that TR-AM self-renewal partially contributed to their increased numbers during the fibrotic phase.

We next performed a FACS analysis of lung macrophages and found that the percentage of tdT^+^ TRMs sharply decreased by day 3 but remained largely unchanged thereafter (Fig. 2g, h). Among the labeled TRMs, the percentage of TR-AMs decreased to its lowest point (33.58 ± 3.80%) during the early injury phase and then increased to 68.33 ± 5.08% by day 28, although this value remained below the steady-state level (Fig. 2g, h). In contrast, the proportion of tissue-resident IMs (TR-IMs) peaked on day 7 and subsequently decreased to 19.93 ± 1.50% on day 28, which was still higher than the steady-state level (Fig. 2g, h). Quantification of apoptosis in TRMs (including both TR-AMs and TR-IMs) revealed no significant differences from day 7 to day 28, further indicating that TRM numbers remained relatively constant during this period (Fig. 2f; Supplementary Fig. S5d–f). These findings were corroborated in *CX3CR1-CreER;R26-tdT* mice, in which a similar pattern of TRM reduction at days 3 and 7 followed by a stage of stability thereafter was observed (Supplementary Fig. S1g–l). Taken together, these results highlight that TRMs undergo significant depletion during the acute inflammatory phase of lung injury but remain stable throughout the repair and fibrotic phases, with TR-AM self-renewal contributing to the replenishment of the TRM population in the lungs (Fig. 2i).

### Infiltrating macrophages accumulate during pulmonary fibrosis

To investigate the role of monocytes and MDMs in lung inflammation and fibrosis, we utilized the *Ms4a3-CreER* mouse model^33^, which allows the specific labeling and tracing of monocytes. *Ms4a3-CreER* mice were crossed with *R26-tdT* reporter mice, and following Tam administration, we examined the monocytes labeling efficiency and specificity (Supplementary Fig. S6a, b). There was no detectable leakiness in the labeling of monocytes in the lungs, blood, and BM without Tam treatment (Supplementary Fig. S6c, d, g, i). Additionally, immunostaining of blood samples for CD68, tdT, and Ly6C revealed that nearly all Ly6C^+^ monocytes were tdT-positive but CD68-negative (Supplementary Fig. S6e). FACS analysis of blood and BM further confirmed the high efficiency and specificity of targeting monocytes in this mouse line (Supplementary Fig. S6f). During homeostasis, a small fraction of lung macrophages (2.63 ± 0.38%) originated from circulating monocytes, demonstrating the specificity of *Ms4a3-CreER* for targeting monocytes and MDMs (Supplementary Fig. S6h). Thus, the *Ms4a3-CreER* mouse line is an effective tool for investigating MDMs in the lungs (Supplementary Fig. S6j).

**Fig. 3:**
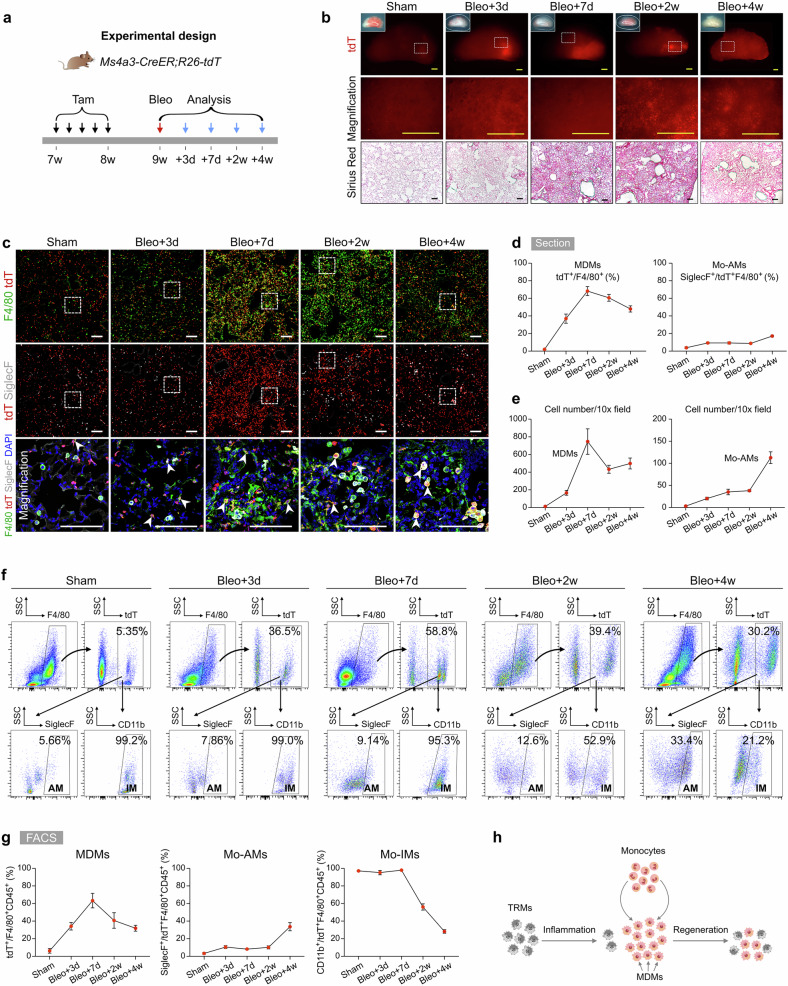
MDMs significantly increased following bleomycin-induced injury. **a** Schematic diagram showing the experimental design. Tam tamoxifen, Bleo bleomycin. **b** Whole-mount bright field, epifluorescence, and Sirius red images of lungs after bleomycin treatment. The boxed region is magnified. **c** Immunostaining for tdT, F4/80, and SiglecF in lung tissue sections. The boxed region is magnified. White arrowheads indicate MDMs. **d** Quantitative analysis of the percentage of F4/80^+^ macrophages expressing tdT (left) and the percentage of alveolar macrophages among labeled tdT^+^ macrophages (right) in immunostained lung sections. Data are presented as the mean ± SEM; n = 5 mice per group. **e** Quantification of the numbers of MDMs (tdT^+^F4/80^+^) and Mo-AMs (tdT^+^SiglecF^+^) in each 10× field from immunostained lung sections. Data are presented as the mean ± SEM; *n* = 5 mice per group. **f**, **g** Flow cytometric and quantification analyses of the percentage of tdT^+^ cells among CD45^+^F4/80^+^ macrophages (MDMs), the percentage of SiglecF^+^ alveolar macrophages among tdT^+^ macrophages (Mo-AMs), and the percentage of CD11b^+^ interstitial macrophages among tdT^+^ macrophages (Mo-IMs) from the lungs. Data are presented as the mean ± SEM; *n* = 5 mice per group. **h** Cartoon image showing the dynamic changes in the number of recruited macrophages in the lungs after bleomycin-induced injury. MDMs monocyte-derived macrophages, Mo-AMs monocyte-derived alveolar macrophages, Mo-IMs monocyte-derived interstitial macrophages. Scale bars, yellow, 1 mm; white and black, 100 µm. Each image is representative of five individual samples.

We collected lung samples from Tam-treated *Ms4a3-CreER;R26-tdT* mice on days 3, 7, 14, and 28 following bleomycin-induced lung injury to track their infiltration and fate during inflammation and fibrosis (Fig. 3a). Whole-mount fluorescence images revealed a low baseline presence of tdT^+^ cells in healthy lungs but significantly increased tdT^+^ signals in injured lungs, which correlated with the extent of tissue damage (Fig. 3b). Sirius red staining of lung sections revealed marked fibrosis, especially at 7 and 14 days postinjury (Fig. 3b). Immunostaining for tdT, F4/80, and SiglecF revealed that tdT^+^ cells were sparsely distributed in the sham group and were mainly Mo-IMs (Fig. 3c, d). During the early stage of lung inflammation, a large influx of tdT^+^ monocytes was observed at injury sites, where these cells began to differentiate into macrophages. By day 7 postinjury, a significant number of tdT^+^ cells accumulated in fibrotic areas, and many of these cells differentiated into macrophages, with a small subset becoming monocyte-derived AMs (Mo-AMs), marked by tdT^+^SiglecF^+^F4/80^+^, and the majority becoming monocyte-derived IMs (Mo-IMs), marked by tdT^+^SiglecF^–^F4/80^+^ (Fig. 3c, e).

As the injury progressed, particularly on day 14, the number of MDMs decreased, whereas the proportion of Mo-AMs in the injury areas increased. By day 28 postinjury, almost half of the macrophages in the lungs were MDMs, with a notable increase in the number of Mo-AMs (Fig. 3c–e). FACS analysis further supported these findings, as the number of MDMs increased during the inflammatory phase but decreased during the lung regeneration phase (Fig. 3f, g). Specifically, the percentage of Mo-IMs sharply decreased from 97.96 ± 0.33% at the peak of inflammation to 28.23 ± 2.04% during recovery, whereas the proportion of Mo-AMs significantly increased, suggesting that some Mo-IMs may convert into Mo-AMs as the lung heals (Fig. 3f, g). Overall, these results reveal that circulating monocytes rapidly infiltrate sites of lung injury, where they primarily differentiate into interstitial macrophages during the inflammatory phase and contribute to alveolar macrophages during the fibrotic phase. This process highlights the dynamic roles of MDMs in both lung inflammation and repair, with the potential conversion of Mo-IMs to Mo-AMs as fibrosis progresses and lung regeneration occurs (Fig. 3h).

### Macrophage heterogeneity during lung injury and repair

**Fig. 4:**
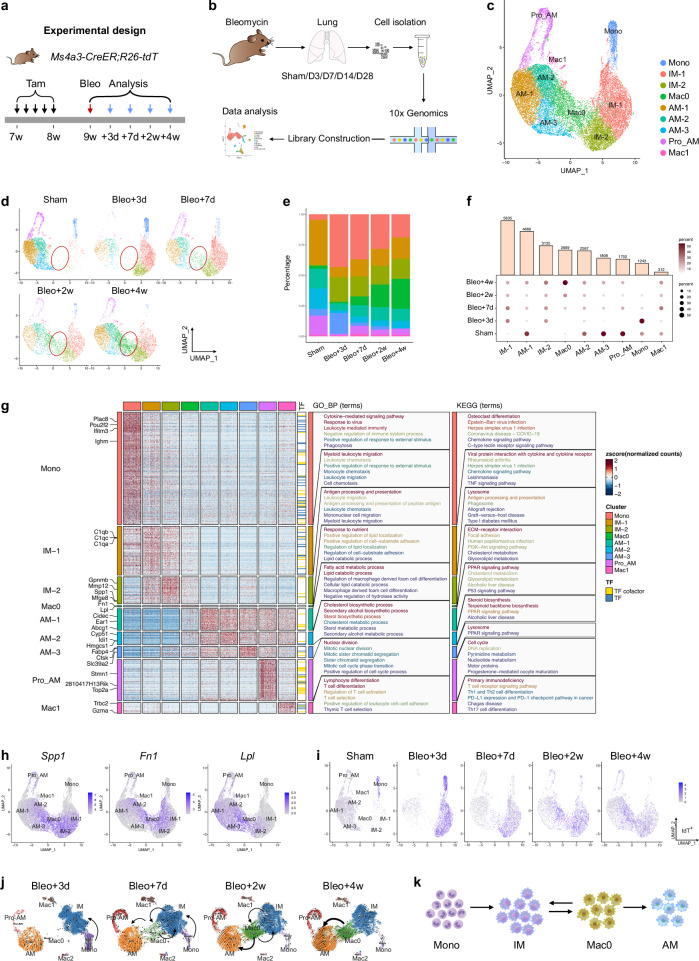
Single-cell landscape of bleomycin-treated lungs. **a** Schematic diagram showing the experimental design. Tam tamoxifen, Bleo bleomycin. **b** Flow chart showing the experimental process. **c** UMAP visualization of macrophage-related cell populations in the mouse lung. **d** UMAP plots indicating the appearance of Mac0 cells on day 7 postinjury. **e** Bar plot representation of the relative frequency of different macrophage subclusters at different time points. **f** Dot plot showing the percentage of each macrophage-related cluster contributing at different time points. The total percentage of one cluster is 100%. **g** Heatmap showing the results of the GO and KEGG pathway analyses of macrophage-related cell populations. **h** FeaturePlot showing the highly expressed genes characteristic of the Mac0 cluster. **i** UMAP projection of labeled monocytes (tdT^+^) visualized separately at different time points. **j** UMAP colored by identified clusters with trajectories inferred from RNA velocity. The black arrows represent the calculated velocity trajectories. The Mac2 cluster resulted from a separate reclustering based on spliced and unspliced RNA counts required for RNA velocity analysis, which differed from the primary analysis using total gene expression. **k** Cartoon image showing the cell fate transition from monocytes to alveolar macrophages. Mono monocytes, IMs interstitial macrophages, AMs alveolar macrophages.

To comprehensively understand the macrophage dynamics in bleomycin-injured lungs, we performed a time series of scRNA-seq analyses. Immune cells were isolated from PBS-treated and bleomycin-injured lungs at 3, 7, 14, and 28 days posttreatment (Fig. 4a, b). A total of 40,006 cells (sham, 8416; bleomycin group: day 3, 7646; day 7, 7956; day 14, 6762; day 28, 9226) passed quality control and were categorized into 11 clusters based on the expression of representative marker genes (Supplementary Fig. S7a). The major immune cell clusters included macrophages (*Itgax*), monocytes (*Csf1r*, *Itgam*), neutrophils (*Clec4e*), T cells (*Il7r*), B cells (*Cd79a*), dendritic cells (*Bst2*), and NK cells (*Klrb1c*) (Supplementary Fig. S7b). To further explore the dynamic changes within each cluster, we examined single-cell transcriptional profiles at individual time points (Supplementary Fig. S7c). Among the immune cell clusters, macrophages accounted for the greatest proportion, and their transcriptional profiles changed significantly during the course of lung injury and repair (Supplementary Fig. S7d, e). The proportion of macrophages decreased after injury but recovered during the fibrotic stage, which was consistent with the lineage tracing results (Fig. 2). Additionally, the percentage of monocytes increased markedly during the early inflammatory phase but gradually decreased during lung repair (Supplementary Fig. S7e).

To gain a more detailed understanding of the transition from homeostasis to the postbleomycin response in macrophages, we aggregated and subclustered macrophages and monocytes from both steady-state and injured samples. Nine subclusters were identified, which could be classified into three macrophage subtypes: AM, IM, and a novel population termed Mac0 (Fig. 4c). In steady-state lungs, the majority of the macrophages were AMs, with low proportions of IMs and a negligible Mac0 population. By day 3 postinjury, a large influx of circulating monocytes infiltrated the lungs, leading to a significant increase in IMs and a decrease in AMs. Simultaneously, the Mac0 cluster began to emerge (Fig. 4d). One week later, Mac0 cells continued to expand and became increasingly involved in lung repair during the fibrotic phase. By day 28 postinjury, the percentage of AMs had partially recovered, while the percentage of IMs had decreased. The number of monocytes remained low during the lung recovery stage (Fig. 4d–f).

Further bioinformatic analysis revealed that the Mac0 cluster exhibited a unique transcriptional profile marked by the upregulation of lysosomal and antigen presentation genes (e.g., *Ctsb*, *H2-Aa*, and *Cd74*), along with the upregulation of genes related to lipid metabolism (e.g., *Lpl* and *Cyp51*) and extracellular matrix interaction (e.g., *Fn1* and *Cd44*). Compared with AMs, Mac0 cells showed reduced alveolar identity but increased injury-responsive functions; compared with monocytes, Mac0 cells presented downregulated expression of inflammatory and migratory genes, indicating a functional transition toward tissue repair. These features positioned Mac0 cells as a transitional, injury-specific subset that supported debris clearance, immunomodulation, and matrix remodeling during lung injury (Fig. 4g; Supplementary Fig. S8a). FeaturePlot and Violin plot analyses revealed that cells within the Mac0 cluster highly expressed the genes *Spp1*, *Fn1*, and *Lpl* (Fig. 4h; Supplementary Fig. S8b).

To better understand the relationships among monocytes, IMs, Mac0 cells, and AMs during lung injury and repair, we performed an RNA velocity analysis on the genetically labeled tdT^+^ cells from *Ms4a3-CreER;R26-tdT* mice (Fig. 4i). This analysis revealed that a subset of tdT^+^ monocytes differentiated into IMs by day 3 postinjury, followed by a clear developmental progression from IMs to Mac0 cells by day 7. Two weeks after injury, labeled tdT^+^ cells were distributed among the IM, Mac0, and AM cell clusters. Four weeks later, the tdT^+^ cells were more concentrated in the AM cluster. These findings suggest that a subset of newly generated AMs arises from IMs via Mac0 cells. To investigate and model this potential cell trajectory, we analyzed the UMAP projection of monocytes and macrophages at various time points postinjury, which revealed a differentiation pathway from monocytes to IMs, then to Mac0 cells and finally toward AMs (Fig. 4j). By day 7 postinjury, Mac0 cells contributed to both the IM and AM populations, but by week 4, Mac0 cells contributed only to AMs.

Pathway overrepresentation analysis using the Kyoto Encyclopedia of Genes and Genomes (KEGG) database indicated that Notch and Wnt/β-catenin signaling were highly enriched in the Mac0 cluster, which is consistent with their activity in AMs (Supplementary Fig. S8c). In terms of transcription, compared with both AMs and IMs, Mac0 cells represented a unique transitional state with a moderate inflammatory signature (Supplementary Fig. S8d–f). Mac0 cells displayed low CD11b (*Itgam*), intermediate SiglecF, and uniform CD11c (*Itgax*) expression, distinguishing them from previously described populations (Supplementary Fig. S8g). Moreover, comparative transcriptomic profiling revealed that the IM1 subcluster exhibited a pronounced proinflammatory and chemotactic phenotype compared with that of the IM2 cluster (Supplementary Fig. S8h).

In summary, we utilized scRNA-seq to characterize the heterogeneity of macrophages after bleomycin-induced lung injury over time and identified a novel transitional macrophage subcluster, Mac0 cells, which originate from monocytes and differentiate into AMs (Fig. 4k).

### Genetic ablation of infiltrating macrophages alleviates pulmonary fibrosis

**Fig. 5:**
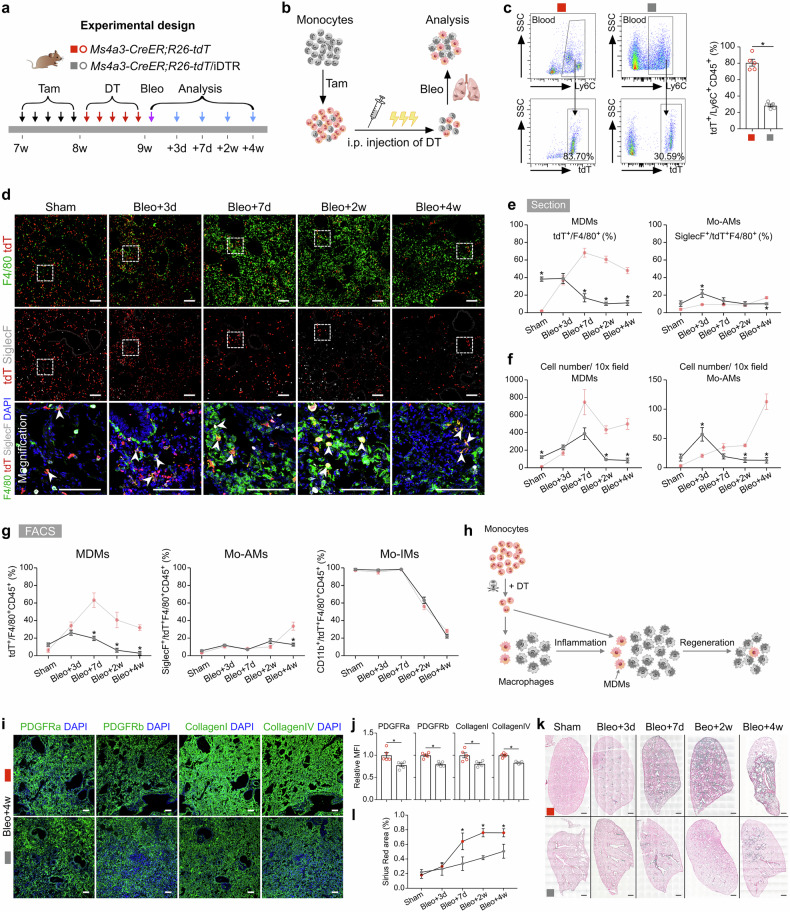
Depletion of monocytes alleviates pulmonary fibrosis after bleomycin treatment. **a** Schematic diagram showing the experimental design. Tam, tamoxifen; DT, diphtheria toxin; Bleo, bleomycin. **b** Schematic diagram illustrating the working process. **c** Flow cytometric and quantification analysis of the percentage of tdT^+^ cells among CD45^+^Ly6C^+^ monocytes from blood. Data are presented as the mean ± SEM; *n* = 5 mice per group. **d** Immunostaining for tdT, F4/80 and SiglecF in tissue sections of *Ms4a3-CreER;R26-tdT/iDTR* lungs. The boxed region is magnified. White arrowheads indicate MDMs. **e** Quantitative analysis of the percentage of F4/80^+^ macrophages expressing tdT (MDMs) and the percentage of Mo-AMs in labeled tdT^+^ macrophages from immunostained lung sections of *Ms4a3-CreER;R26-tdT/iDTR* mice. The results of the control experiment were the same as those in Fig. 3d. Data are presented as the mean ± SEM; *n* = 5 mice per group. **f** Quantification of the numbers of MDMs (tdT^+^F4/80^+^) and Mo-AMs (tdT^+^SiglecF^+^) in each 10× field from immunostained lung sections of *Ms4a3-CreER;R26-tdT/iDTR* mice. The results of the control experiment were the same as those in Fig. 3e. Data are presented as the mean ± SEM; *n* = 5 mice per group. **g** Flow cytometric and quantification analyses of the percentage of tdT^+^ cells among CD45^+^F4/80^+^ macrophages (MDMs), the percentage of SiglecF^+^ alveolar macrophages among tdT^+^ macrophages (Mo-AMs), and the percentage of CD11b^+^ interstitial macrophages among tdT^+^ macrophages (Mo-IMs) from the lungs. The results of the control experiment were the same as those in Fig. 3g. Data are presented as the mean ± SEM; *n* = 5 mice per group. **h** Cartoon image showing the dynamic changes in the number of recruited macrophages in *Ms4a3-CreER;R26-tdT/iDTR* lungs after treatment with bleomycin. MDMs, monocyte-derived macrophages; Mo-AMs, monocyte-derived alveolar macrophages; Mo-IMs, monocyte-derived interstitial macrophages. **i** Immunostaining for PDGFRa, PDGFRb, collagen I, and collagen IV in lung tissue sections. **j** Quantitative analysis of the relative mean fluorescence intensity (MFI) of PDGFRa, PDGFRb, collagen I, and collagen IV per 10× field of lung sections. The data are presented as the mean ± SEM; *n* = 5 mice per group. **k**, **l** Sirius red images and quantification of pulmonary fibrosis after bleomycin treatment. Data are presented as the mean ± SEM; *n* = 5 mice per group. Scale bars, white, 100 µm; black, 1 mm. Each image is representative of five individual samples.

Monocyte-derived macrophages (MDMs) have been shown to drive fibrogenesis in various organs, partly through their interactions with fibroblasts^7,28,34^. To specifically investigate the reparative role of MDMs at different stages following lung injury, we crossed *Ms4a3-CreER;R26-tdT* mice with the inducible diphtheria toxin receptor (DTR) mouse line *R26-iDTR*, allowing for the ablation of monocytes via diphtheria toxin (DT) treatment (Fig. 5a). First, we treated the *Ms4a3-CreER;R26-tdT/iDTR* and *Ms4a3-CreER;R26-tdT* control mice with Tam to efficiently label monocytes with tdT, followed by DT injection, followed by the induction of bleomycin injury (Fig. 5b). FACS analysis revealed a significant reduction in the number of circulating tdT^+^ monocytes in *Ms4a3-CreER;R26-tdT/iDTR* mice, indicating that the majority of tdT^+^ monocytes were successfully ablated (Fig. 5c). Whole-mount fluorescence imaging of the lungs of these mice revealed reduced tdT^+^ signals in fibrotic regions (Supplementary Fig. S9a). Additionally, Sirius red staining of lung sections revealed a reduced fibrotic response in the monocyte-depleted groups compared with the control groups (Supplementary Fig. S9b).

Immunostaining for tdT, F4/80, and SiglecF in lung sections revealed a markedly greater number of tdT^+^ macrophages at homeostasis in *Ms4a3-CreER;R26-tdT/iDTR* mice than in *Ms4a3-CreER;R26-tdT* controls after DT treatment (Fig. 5d). Quantitative analysis further revealed a significantly increased proportion of MDMs in monocyte-ablated mice without injury, indicating that monocyte depletion during homeostasis triggered a strong immune response (Fig. 5e, g). Following bleomycin injury, the decrease in the number of circulating monocytes led to a significant decrease in the number of MDMs, particularly Mo-AMs, on day 28 (Fig. 5e–g). By day 7 postinjury, only 19.75 ± 2.57% of the MDMs were present at the injury site, whereas this percentage was 63.29 ± 8.25% in the control lungs (Fig. 5e compared with Fig. 3d). During the fibrotic phase, very few MDMs were detected in the injured area (Fig. 5e, g). Interestingly, the ablation of monocytes had little effect on the percentage of Mo-IMs throughout the injury and recovery process (Fig. 5g). Overall, these results demonstrated that monocytes and MDMs could be specifically and effectively depleted in *Ms4a3-CreER;R26-tdT/iDTR* mice during the inflammatory and fibrotic phases (Fig. 5h).

Next, we explored the role of monocytes in pulmonary inflammation and fibrosis. Quantitative real-time PCR (qRT-PCR) revealed that the deletion of circulating monocytes led to a reduction in the expression of anti-inflammatory genes such as *Arg1* and *Chil3*, while the expression of the proinflammatory genes *Il6 and Il1β* was significantly upregulated, indicating that monocyte ablation aggravated inflammation in the early stage after injury (Supplementary Fig. S9c). Furthermore, the expression of profibrotic and fibroblast markers, including *Fn1*, *Col3a1*, *Pdgfra*, and *Pdgfrb*, was markedly reduced in the monocyte-depleted groups during the recovery phase, suggesting that bleomycin-induced fibrosis was more severe in control lungs (Supplementary Fig. S9c). Immunostaining for PDGFRa, PDGFRb, collagen I, and collagen IV in lung sections revealed reduced protein expression levels in monocyte-depleted mice after bleomycin treatment (Fig. 5i, j). Histological studies further confirmed that monocyte ablation significantly reduced the extent of pulmonary fibrosis during the progressive fibrotic phase (Fig. 5k, l).

Notably, neutrophils were also affected by DT treatment in *Ms4a3-CreER;R26-tdT/iDTR* mice. However, in our model, neutrophils, which were primarily associated with acute inflammation rather than fibrosis, exhibited early infiltration that peaked within the first week after injury but decreased sharply during the fibrotic phase (Supplementary Fig. S9d). Temporal analysis revealed no significant difference in lung neutrophil levels during key inflammatory and fibrotic stages following depletion. These kinetic patterns suggested that the reduced fibrosis was more likely due to the depletion of MDMs and Mo-AMs than to the loss of neutrophils.

Taken together, these results suggest that the depletion of circulating monocytes affects lung macrophage populations in both physiological and pathological states, reducing fibroblast accumulation and alleviating fibrosis following lung injury.

### Monocyte-specific Notch knockout attenuates pulmonary fibrosis

**Fig. 6:**
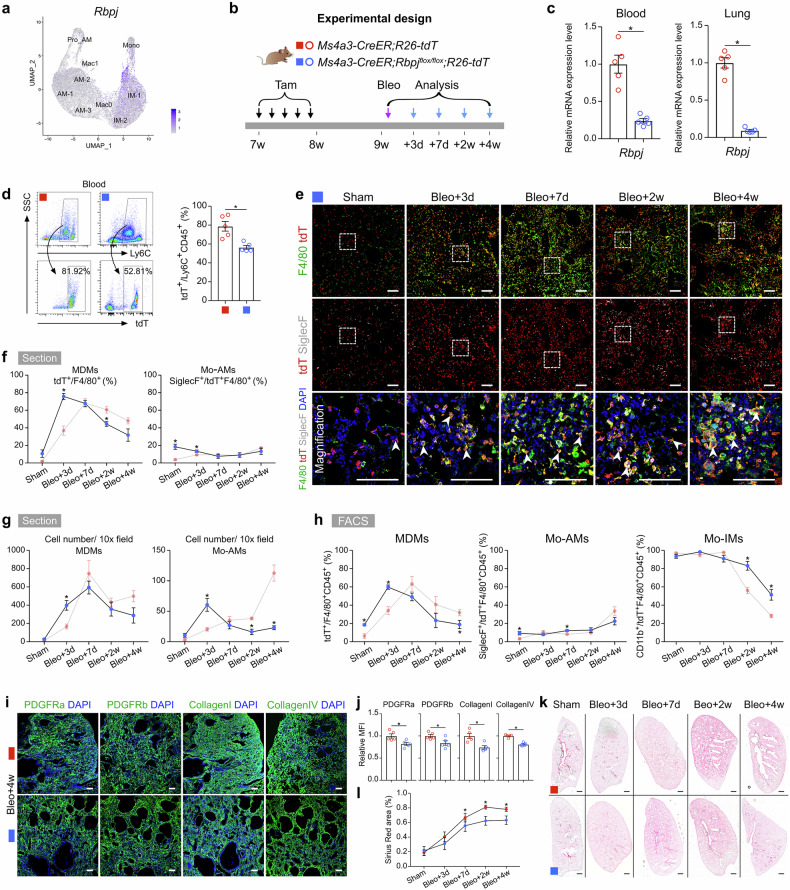
Monocyte-specific *Rbpj* knockout blocks monocyte differentiation into alveolar macrophages and reduces pulmonary fibrosis. **a** Feature plots illustrating *Rbpj* expression in macrophage-related clusters. **b** Schematic diagram showing the experimental design. Tam tamoxifen, Bleo bleomycin. **c** qRT-PCR analysis showing the knockout efficiency of *Rbpj* in tdT^+^ cells from the blood and lungs. Data are presented as the mean ± SEM; *n* = 5 mice per group. **d** Flow cytometric and quantification analysis of the percentage of tdT^+^ cells among CD45^+^Ly6C^+^ monocytes from blood. Data are presented as the mean ± SEM; *n* = 5 mice per group. **e** Immunostaining for tdT, F4/80 and SiglecF in tissue sections of *Ms4a3-CreER;Rbpj*^*flox/flox*^*;R26-tdT* lungs. The boxed region is magnified. White arrowheads indicate MDMs. **f** Quantitative analysis of the percentage of F4/80^+^ macrophages expressing tdT (MDMs) and the percentage of Mo-AMs in labeled tdT^+^ macrophages from immunostained lung sections from *Ms4a3-CreER;Rbpj*^*flox/flox*^*;R26-tdT* mice. The results of the control experiment were the same as those in Fig. 3d. Data are presented as the mean ± SEM; *n* = 5 mice per group. **g** Quantification of the numbers of MDMs (tdT^+^F4/80^+^) and Mo-AMs (tdT^+^SiglecF^+^) in each 10× field of immunostained lung sections from *Ms4a3-CreER;Rbpj*^*flox/flox*^*;R26-tdT* mice. The results of the control experiment were the same as those in Fig. 3e. Data are presented as the mean ± SEM; n = 5 mice per group. **h** Flow cytometric and quantification analyses of the percentage of tdT^+^ cells among CD45^+^F4/80^+^ macrophages (MDMs), the percentage of SiglecF^+^ alveolar macrophages among tdT^+^ macrophages (Mo-AMs), and the percentage of CD11b^+^ interstitial macrophages among tdT^+^ macrophages (Mo-IMs) from the lungs. The results of the control experiment were the same as those in Fig. 3g. Data are presented as the mean ± SEM; *n* = 5 mice per group. **i** Immunostaining for PDGFRa, PDGFRb, collagen I, and collagen IV in lung tissue sections. **j** Quantitative analysis of the relative mean fluorescence intensity (MFI) of PDGFRa, PDGFRb, collagen I, and collagen IV per 10× field of lung sections. Data are presented as the mean ± SEM; *n* = 5 mice per group. **k**, **l** Sirius red images and quantification of pulmonary fibrosis after bleomycin treatment. Data are presented as the mean ± SEM; *n* = 5 mice per group. Scale bars, white, 100 µm; black, 1 mm. Each image is representative of five individual samples.

RBP-J-mediated Notch signaling has been demonstrated to be a crucial regulator of monocyte differentiation and function. RBP-J, known as the master nuclear mediator of canonical Notch signaling^35^, also plays a pivotal role in monocyte cell fate decisions^36–38^. Our scRNA-seq analysis indicated that Notch signaling was markedly activated in the AM and Mac0 clusters during lung repair and regeneration (Fig. 6a; Supplementary Fig. S8c). We generated monocyte-specific *Rbpj* knockout mice by crossing *Rbpj*^*flox/flox*^ mice with *Ms4a3-CreER;R26-tdT* mice (Fig. 6b). Bleomycin injury was induced 1 week after Tam treatment (Fig. 6b). qRT-PCR analysis confirmed the efficient knockout of *Rbpj* in sorted tdT^+^ cells from both blood and lungs (Fig. 6c). FACS analysis revealed that the conditional knockout of *Rbpj* in monocytes resulted in a reduction in the number of circulating monocytes (Fig. 6d). Immunostaining for tdT, F4/80, and SiglecF in lung sections revealed that on day 3 postinjury, Rbpj deficiency resulted in an increase in the number of MDMs, which subsequently decreased by day 7 (Fig. 6e–g). Although the number of Mo-AMs was significantly increased on day 3 after injury, it sharply decreased thereafter (Fig. 6g). This reduction in the number of Mo-AMs during the fibrotic phase suggested defective differentiation of Mo-IMs into Mo-AMs (Fig. 6f, g). FACS analysis further confirmed that in the absence of injury, the percentage of MDMs increased to 18.55 ± 1.06% in *Rbpj* knockout mice compared with 6.18 ± 2.64% in wild-type mice (Fig. 6h). Additionally, the elevated proportion of Mo-IMs on day 28 in *Rbpj* knockout mice suggested that Notch signaling may promote the differentiation of Mo-IMs into Mo-AMs (Fig. 6h).

The expression of signature genes associated with the M2 phenotype, such as *Arg1* and *Chil3*, was significantly upregulated in *Rbpj* knockout mice. In contrast, the gene expression of proinflammatory cytokines and chemokines, including *Il6* and *Il1β*, was unchanged or downregulated (Supplementary Fig. S10). Immunostaining revealed reduced expression of PDGFRa, PDGFRb, collagen I, and collagen IV and a decreased degree of fibrosis, as assessed by Sirius red staining, in the lungs of *Rbpj* knockout mice following bleomycin treatment (Fig. 6i–l). qRT-PCR analysis further validated the reduced mRNA levels of profibrotic and fibrosis-related genes, including *Fn1*, *Pdgfra*, and *Pdgfrb*, in *Rbpj* knockout mice after injury (Supplementary Fig. S10). To determine whether the conditional knockout of *Rbpj* in monocytes affects the function of Mo-AMs and Mo-IMs, we isolated tdT^+^ Mo-AMs and Mo-IMs from the lung tissues of knockout mice and assessed their phagocytic capacity and polarization status (Supplementary Fig. S11). In vitro functional analysis revealed significantly impaired phagocytosis in both Mo-AMs and Mo-IMs. Additionally, the loss of *Rbpj* promoted a shift toward M2-like polarization in both subsets.

Collectively, these findings demonstrate that monocyte-specific *Rbpj* deficiency not only impairs the differentiation of monocytes into AMs but also disrupts key functional properties of MDMs, ultimately attenuating bleomycin-induced lung inflammation and fibrosis.

### Loss of Wnt/β-catenin signaling in monocytes exacerbates pulmonary fibrosis

**Fig. 7:**
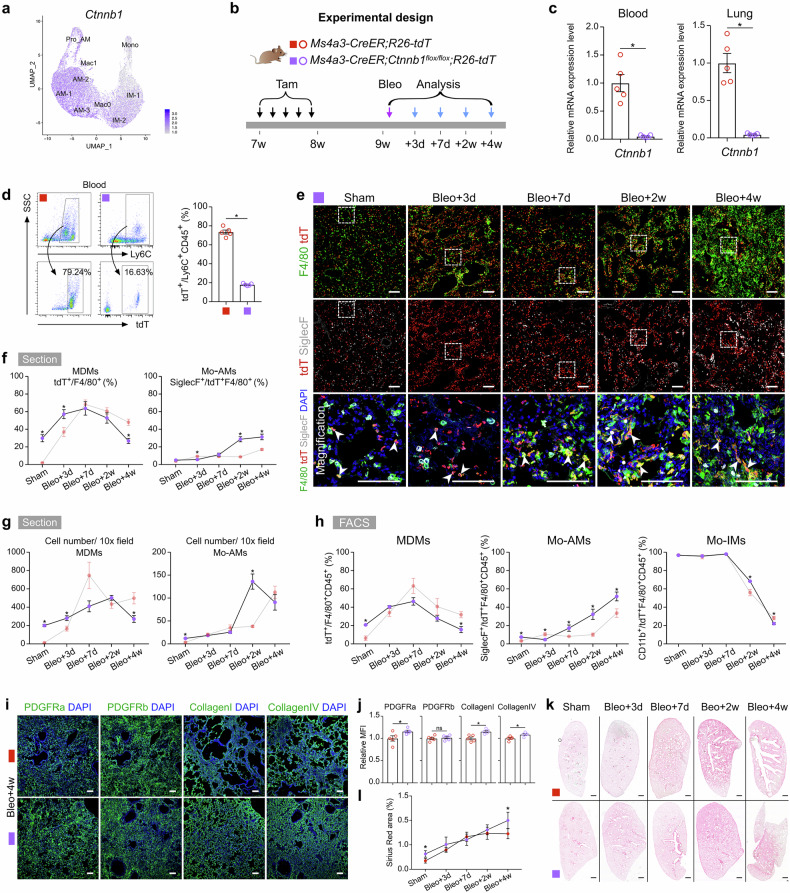
Monocyte-specific *Ctnnb1* knockout promotes monocyte differentiation into AMs and contributes to persistent fibrosis after bleomycin treatment. **a** Feature plots illustrating *Ctnnb1* expression in macrophage-related clusters. **b** Schematic showing the experimental design. Tam tamoxifen, Bleo bleomycin. **c** qRT-PCR analysis showing the knockout efficiency of *Ctnnb1* in tdT^+^ cells from the blood and lungs. Data are presented as the mean ± SEM; *n* = 5 mice per group. **d** Flow cytometric and quantification analyses of the percentage of tdT^+^ cells among CD45^+^Ly6C^+^ monocytes from blood. Data are presented as the mean ± SEM; *n* = 5 mice per group. **e** Immunostaining for tdT, F4/80 and SiglecF in tissue sections of *Ms4a3-CreER;Ctnnb1*^*flox/flox*^*;R26-tdT* lungs. The boxed region is magnified. White arrowheads indicate monocyte-derived macrophages (MDMs). **f** Quantitative analysis of the percentage of F4/80^+^ macrophages expressing tdT (MDMs) and the percentage of Mo-AMs among labeled tdT^+^ macrophages in immunostained lung sections from *Ms4a3-CreER;Ctnnb1*^*flox/flox*^*;R26-tdT* mice. The results of the control experiment were the same as those in Fig. 3d. Data are presented as the mean ± SEM; *n* = 5 mice per group. **g** Quantification of the numbers of MDMs (tdT^+^F4/80^+^) and Mo-AMs (tdT^+^SiglecF^+^) in each 10× field of immunostained lung sections from *Ms4a3-CreER;Ctnnb1*^*flox/flox*^*;R26-tdT* mice. The results of the control experiment were the same as those in Fig. 3e. Data are presented as the mean ± SEM; *n* = 5 mice per group. **h** Flow cytometric and quantification analyses of the percentage of tdT^+^ cells among CD45^+^F4/80^+^ macrophages (MDMs), the percentage of SiglecF^+^ alveolar macrophages among tdT^+^ macrophages (Mo-AMs), and the percentage of CD11b^+^ interstitial macrophages among tdT^+^ macrophages (Mo-IMs) from the lungs. The results of the control experiment were the same as those in Fig. 3g. Data are presented as the mean ± SEM; *n* = 5 mice per group. **i** Immunostaining for PDGFRa, PDGFRb, collagen I, and collagen IV in lung tissue sections. The boxed region is magnified. **j** Quantitative analysis of the relative mean fluorescence intensity (MFI) of PDGFRa, PDGFRb, collagen I, and collagen IV per 10× field of lung sections. Data are presented as the mean ± SEM; *n* = 5 mice per group. **k**, **l** Sirius red images and quantification analyses of pulmonary fibrosis after bleomycin treatment. Data are presented as the mean ± SEM; *n* = 5 mice per group. Scale bars, white, 100 µm; black, 1 mm. Each image is representative of five individual samples.

Previous studies have indicated that Wnt/β-catenin signaling plays a critical role in the differentiation of monocytes into macrophages^39–41^. Our scRNA-seq data revealed that Wnt/β-catenin signaling was active in AM and Mac0 clusters (Fig. 7a). β-Catenin, encoded by the *Ctnnb1* gene, is a key effector downstream of canonical Wnt signaling^42^. To further investigate the specific roles of Wnt/β-catenin in the recruitment of macrophages during lung repair and regeneration, we crossed *Ctnnb1*^*flox/flox*^ mice with *Ms4a3-CreER;R26-tdT* mice to specifically disrupt Wnt/β-catenin signaling in monocytes (Fig. 7b). The knockout efficiency in blood and lung tissues exceeded 95% (Fig. 7c). We observed a significant reduction in the number of monocytes in *Ctnnb1* knockout mice in the absence of injury (Fig. 7d). Immunostaining for tdT, F4/80, and SiglecF in lung sections, along with quantification analysis, revealed a greater percentage of MDMs at homeostasis in *Ctnnb1* knockout mice (Fig. 7e–g).

Following injury, the percentage of MDMs increased to 63.65 ± 7.61% by day 7 in *Ctnnb1* knockout mice, similar to that in wild-type controls. However, by day 28, this percentage decreased to 26.78 ± 2.85%, which was significantly lower than that in wild-type mice (Fig. 7e–g). During the progression of fibrosis, a significant increase in the number of recruited Mo-AMs was observed in *Ctnnb1*-knockout mice (Fig. 7e–g). FACS analysis also revealed an increase in the percentage of MDMs in uninjured lungs, and a high level of Mo-AMs was maintained until day 28 after bleomycin treatment. However, absolute cell quantification indicated that the apparent increase in the proportion of Mo-AMs on day 28 was primarily due to a reduction in total MDMs rather than a substantial increase in absolute Mo-AM numbers (Supplementary Fig. S12). Notably, the percentage of Mo-IMs remained unaffected throughout the entire process in *Ctnnb1*-knockout mice (Fig. 7h).

The expression of M1 macrophage cytokines, such as *Il6* and *Il1β*, which are associated with proinflammatory responses, was significantly greater on days 3 and 7 postinjury in *Ctnnb1* knockout mice than in wild-type control mice. Moreover, the expression of M2 macrophage-related genes, such as *Arg1* and *Chil3*, did not significantly change, suggesting that Wnt/β-catenin blockade promoted M1 macrophage polarization under inflammatory stimulation (Supplementary Fig. S10). Moreover, 4 weeks after bleomycin treatment, there was a marked increase in lung fibrosis in *Ctnnb1*-knockout mice, as assessed by anti-PDGFRa, anti-PDGFRb, anti-collagen I, and anti-collagen IV immunofluorescence staining and Sirius red staining of lung sections (Fig. 7i–l). Consistently, qRT-PCR analysis revealed elevated mRNA expression of profibrotic genes, such as *Col3a1*, in β-catenin-deficient mice (Supplementary Fig. S10). Furthermore, isolated tdT^+^ Mo-AMs and Mo-IMs from *Ctnnb1* monocyte-specific knockout mice exhibited impaired phagocytic ability along with distinct alterations in polarization, specifically, reduced M1 markers in Mo-AMs and increased M1 coupled with decreased M2 markers in Mo-IMs (Supplementary Fig. S11). These results indicate that the conditional knockout of *Ctnnb1* disrupted both the phagocytic function and the polarization of MDMs.

Overall, the loss of Wnt/β-catenin signaling in monocytes significantly increased the number of MDMs at homeostasis and promoted the differentiation of Mo-IMs into Mo-AMs during recovery. This dysregulated differentiation likely contributed to severe pulmonary fibrosis.

## Discussion

The lung damage caused by pulmonary fibrosis, the leading cause of mortality in patients with lung disease, cannot be fully repaired. Conventional therapies focus primarily on slowing the rate of fibrosis. Understanding the cellular and molecular mechanisms underlying these conditions is crucial for developing more effective treatments. Macrophages play an indispensable role in driving lung inflammation, regulating fibrosis, and mediating tissue repair and regeneration. Two major sources of macrophages involved in lung injury and repair are TRMs and MDMs. A comprehensive understanding of the heterogeneous origins of macrophages and their dynamic changes is essential not only for discerning the functions of these cells in health and disease but also for identifying novel therapeutic targets. However, the dynamic responses of macrophages from different cell lineages during the progression of lung injury remain unclear.

In this study, we utilized genetic lineage tracing, scRNA-seq, genetic cell ablation, and gene knockout experiments to elucidate the cellular dynamics and functions of macrophages from different origins in the lung during homeostasis, injury, and repair. Our results demonstrate that in the absence of injury, lung macrophages self-maintained local levels, with approximately 5% of the cells originating from circulating monocytes. During the inflammatory phase shortly after bleomycin injury, the number of lung TRMs, particularly TR-AMs, significantly decreased, which coincided with increases in the numbers of MDMs and Mo-IMs. We also found that enhanced proliferation of resident macrophages, particularly TR-AMs, occurred during the initial stages of inflammation. These findings suggest that the proliferation of resident macrophages is involved in anti-inflammatory processes^14^. In the repair and regeneration phase, lung macrophages were predominantly composed of MDMs. Notably, scRNA-seq and pseudotime trajectory analyses revealed CCR2^+^Ly6C^high^ inflammatory monocytes as the primary source of IMs, with both *Ccr2* and *Ly6c* expression peaking during the monocyte-to-IM transition (Supplementary Fig. S13). Moreover, during this stage, more Mo-IMs differentiated into Mo-AMs, resulting in an increased proportion of Mo-AMs. Previous studies have indicated that recruited AMs undergo apoptosis and are removed from the airspace during the resolution of inflammation^43,44^. Moreover, when resident AMs are ablated by clodronate or radiation, recruited AMs can fill and maintain the niche^45^. RNA sequencing analysis revealed that the transcriptional profiles of TRMs and MDMs, particularly TR-AMs and Mo-AMs, significantly differed in gene expression during the development of bleomycin-induced pulmonary fibrosis^46,47^. Nevertheless, Mo-AMs clearly correlated with the severity of fibrosis. The necroptosis of Mo-AMs was associated with reduced fibrosis, whereas the deletion of TR-AMs had no discernible effect on fibrosis severity^46^. Our findings consistently demonstrate that the number of MDMs, particularly Mo-AMs, was significantly reduced and that the lung exhibited less pulmonary fibrosis following monocyte ablation.

Previous studies have indicated that targeting the pathways essential for AM differentiation may be an effective strategy for preventing pulmonary fibrosis^40,48,49^. Our study verified that the Notch and Wnt/β-catenin signaling pathways were activated during monocyte-to-macrophage differentiation. Similar to the monocyte depletion mouse model, the conditional knockout of *Rbpj* in monocytes led to a reduction in the number of MDMs and decreased the differentiation of Mo-IMs into Mo-AMs, accompanied by reduced lung fibrosis. In contrast, in monocyte-specific *Ctnnb1* knockout mice, the number of MDMs increased without injury but decreased significantly after injury compared with that in wild-type controls. Mo-IMs in *Ctnnb1* knockout mice preferentially differentiated into Mo-AMs, exacerbating pulmonary fibrosis. To investigate the potential crosstalk between the Notch and Wnt/β-catenin signaling pathways during the differentiation of monocytes to Mo-AMs, we performed an in-depth analysis of our scRNA-seq data (Supplementary Fig. S14). The results revealed a dynamic and sequential regulatory pattern rather than direct simultaneous crosstalk: Notch signaling plays a key role during the intermediate (IM) transitional stage, whereas Wnt signaling is predominant in later stages to establish and/or maintain the mature Mo-AM phenotype.

It is evident that lung macrophages play pivotal roles in maintaining homeostasis and in the pathogenesis of lung disease. The data presented here, derived from genetic lineage tracing of TRMs and MDMs, highlight their distinct roles during lung inflammation, fibrosis, and repair. With respect to AMs, we and others have demonstrated that TR-AMs and Mo-AMs exhibit a high degree of heterogeneity and dynamic changes in the expression of profibrotic genes during disease progression^50^. However, several questions remain unanswered. For instance, it is still unclear whether Mo-AMs ultimately become indistinguishable from embryonically derived TR-AMs in the context of pulmonary diseases. Studies on IMs are limited, and there is a paucity of knowledge regarding their cell fate and functions following injury. In our studies, we found that TRMs and MDMs played complementary roles in the acute phase after bleomycin-induced injury, with Mo-IMs emerging as key players in promoting lung inflammation. Additionally, several studies have indicated that the metabolic status of TRMs and MDMs varies during disease progression and is correlated with tissue fibrosis^50–52^. During the fibrotic process induced by bleomycin, macrophages undergo metabolic rewiring from lipid metabolism to glucose metabolism. This transition is accompanied by an increase in arginine synthesis and metabolism^50^. These findings were corroborated in an idiopathic pulmonary fibrosis (IPF) mouse model^53^. Recent studies have demonstrated that in the airways, alveolar macrophages are highly dependent on GM-CSF. The cell-specific deletion of GM-CSF in alveolar type 2 (AT2) epithelial cells resulted in impaired development of alveolar macrophages^54,55^. The cytokine TGF-β plays a role in the communication between macrophages and alveolar epithelial cells. The loss of autocrine TGF-β leads to the spontaneous production of proinflammatory cytokines and chemokines by macrophages^55–57^. Further investigation of the interactions between macrophages and the local environment is essential for enhancing our understanding of inflammatory lung disease.

In addition, RNA velocity analysis revealed a newly identified macrophage subpopulation, Mac0, which formed immediately during the acute phase of inflammation and became one of the major macrophage subtypes by day 7. This population expressed genes involved in antigen processing and presentation, biosynthetic processes, metabolic processes, and inflammatory responses. Lineage tracing combined with scRNA-seq confirmed that Mac0 cells originated from monocytes. Cell trajectory analysis further demonstrated that Mac0 cells can differentiate into both IMs and AMs during the inflammatory phase but primarily differentiate into AMs during the fibrotic phase. Genetic ablation of monocytes resulted in a reduction in Mac0 cells, which facilitated the resolution of pulmonary fibrosis. Similarly, using a novel hierarchical clustering approach based on SingleR correlations, Aran et al. identified a disease-related macrophage subgroup with transitional gene expression between monocyte-derived and alveolar macrophages^58^. This specific subgroup, marked as CX3CR1^+^SiglecF^+^ transitional macrophages, was found to localize to the fibrotic niche and displayed profibrotic activity.

In summary, through genetic approaches to specifically label and trace TRMs and MDMs in vivo, we elucidated the dynamic changes in and cell fate transitions of macrophages during lung injury and recovery. Cell ablation and gene manipulation further demonstrated that both TRMs and MDMs are essential for the fibrotic response to lung injury, each of which plays distinct roles throughout lung inflammation (Supplementary Fig. S15). Elucidating the roles of TRMs and MDMs in the development of pulmonary fibrosis is critical for advancing our understanding of lung disease pathophysiology and regeneration, potentially offering new therapeutic strategies for treating this deadly pulmonary disorder.

## Materials and methods

### Mice

All animal protocols performed in this study were approved by the Institutional Animal Care and Use Committee (IACUC) of the Shanghai Institute of Biochemistry and Cell Biology, Center for Excellence in Molecular Cell Science, Chinese Academy of Sciences. The previously described mouse strains used in this study included *CD68-rtTA*, *TetO-Cre*, *R26-tdT*, *Ms4a3-CreER*, *R26-iDTR*, *Rbpj*^*flox/flox*^, *Ctnnb1*^*flox/flox*^, and *CX*_*3*_*CR1-CreER* mice^33,59–61^. The *CD68-rtTA* mouse line was generated by Shanghai Model Organisms Center, Inc., using the CRISPR/Cas9 method. For *CD68-rtTA*, the cDNA encoding rtTA-WPRE-pA was inserted into the *CD68* gene locus between the 5′UTR and exon 1. All the mouse lines were maintained on a C57BL6/129 mixed genetic background. Tamoxifen (Sigma, T5648) was dissolved in corn oil (20 mg/mL) and administered by gavage at 0.2 mg/g body weight at the indicated time points. For doxycycline (Dox, Sigma, D9891) administration, dox was dissolved in mouse drinking water at 2 mg/mL for 1 week. For diphtheria toxin (DT, Sigma, D0564-1MG) administration, DT was dissolved in PBS, and the mice were injected intraperitoneally at 10 ng/g body weight. Both male and female adult mice beginning at 7 weeks of age were included in this study. Mice of the indicated genotypes were assigned randomly to the experimental groups.

### Bleomycin-induced injury

Acute lung injury was induced by intratracheal instillation of bleomycin, as previously described^59^. In detail, bleomycin (Sigma B8416) reagent was dissolved at a concentration of 10 U/mL in sterile PBS (Invitrogen, 10010049) and stored as small aliquots at −80 °C. Before use, 10 U/mL bleomycin was diluted to a working concentration of 1 U/mL with PBS. After the mice were anesthetized with 1% pentobarbital sodium, 2 U/kg bleomycin was pipetted into the cannula. As the mice breathed, bleomycin was inhaled into the lungs. The control mice were treated with PBS.

### Genomic PCR

For genotyping, biopsies of mice were collected and digested in tail lysis buffer (300 μL, 100 mmol/L Tris HCL at pH 7.8, 5 mmol/L EDTA, 0.2% sodium dodecyl sulfate, and 200 mmol/L NaCl) supplemented with proteinase K (5 μL, Roche, 3115852001) at 55 °C overnight. Then, 600 μL absolute EtOH was added for centrifugation at 13,400× *g* for 2 min. The supernatant was discarded, and 600 μL 70% EtOH was added for centrifugation at 13,400× *g* for 2 min. Afterward, the supernatant was discarded, and the precipitate was air-dried. Two hundred microliters of distilled water was added to dissolve the DNA, and the supernatant was used for PCR amplification.

### Cell isolation and flow cytometry

This protocol was designed primarily to target immune cell populations, with a limited number of structural cells. After being euthanized, the mice were perfused with 10 mL of cold PBS through the right ventricle to flush out blood cells in the lung. Then, the mice were inflated through the trachea with 2 mL of digestion solution (0.2 mg/mL collagenase IV, 5% FBS, and 60 U/mL DNase I) in RPMI-1640 media (FBS, Gibco, 10099141; RPMI-1640, Invitrogen, 22400089). The lungs were removed and minced into small pieces in 25 mL of digestion solution for 30 min at 37 °C with shaking and frequent agitation. After digestion, the cells were filtered through a 70-μm strainer and centrifuged at 500× *g* for 5 min at 4 °C, after which the supernatant was discarded. Next, the cells were incubated in 1 mL of red blood cell lysis buffer (eBioscience, 00-4333-57) at room temperature for 5 min. Nine milliliters of cold PBS was added, and the sample was centrifuged at 500× *g* for 5 min at 4 °C, after which the supernatant was discarded. After red blood cell lysis, the cells were washed twice with cold PBS before staining. For blood cells, mouse blood was collected in a heparin-containing cold PBS solution. After being filtered through a 70-μm cell strainer and red blood cell lysis, the cells were washed twice with cold PBS before staining. To obtain bone marrow cells, bone marrow from mouse femurs and tibias was harvested in cold PBS solution. The marrow suspension was broken down by pipetting and filtration through a 70-μm cell strainer. After red blood cells were lysed and washed with PBS, the bone marrow cells were prepared for staining. For immunostaining, the cell pellet was resuspended in 1% Fc block (CD16/CD32, eBioscience, 14-0161-82) in PBS for 5 min at room temperature. Then, the cells were stained with primary antibodies against CD45 APC-eFluor 780 (eBioscience, 47-0451-82, 1:200), F4/80 PE-Cy7 (BioLegend, 123114, 1:200), CD11b APC (eBioscience, 17-0112-81, 1:200), CD11c FITC (eBioscience, 11-0114-82, 1:200), SiglecF FITC (eBioscience, 53-1702-82, 1:200), Ly6C FITC (BD Biosciences, 553104, 1:200), MerTK PE-Cy7 (eBioscience, 25-5751-82, 1:200), CD64 BV711 (BioLegend, 139311, 1:200), and Ly6G APC (eBioscience, 17-9668-80, 1:200) at 4 °C for 30 min. After that, the cells were washed and resuspended in cold PBS and then stained with DAPI (Vector Laboratories, 1:1000) at 4 °C for 5 min before FACS. The cells were analyzed using an Attune NxT flow cytometer (Thermo Fisher Scientific) and sorted by a FACSAria SORP instrument (BD). Data were generated using FlowJo (Tree Star). The gating strategies for flow cytometry data analysis are illustrated in Supplementary Fig. S16.

### Tissue whole-mount fluorescence microscopy

Tissues were dissected and collected in cold phosphate-buffered saline (PBS) and fixed in 4% paraformaldehyde for 1 h at 4 °C. The tissues were then washed several times in PBS and placed on 1% agar in the required orientation for whole-mount bright-field and fluorescence imaging using a Zeiss stereoscope (AxioZoom V16).

### Immunofluorescence (IF) staining and imaging

Immunostaining was performed according to a previously described protocol^59^. After the mice were euthanized, the tissues were washed in cold PBS and fixed in 4% paraformaldehyde (PFA, Sigma, P6148) for no more than 1 h at 4 °C. After fixation, the tissues were rinsed several times in PBS and incubated in 30% sucrose at 4 °C overnight. Once the tissues sank to the bottom, the tissues were embedded in OCT (Sakura, 4583) medium in the required orientation and stored at −80 °C. Cryosections were prepared at a thickness of 10 μm and stored at −20 °C before use. Before staining, the sections were placed in a fume hood to air dry for 1 h and then washed three times in 1× PBS for 5 min each. The sections were subsequently blocked with 5% donkey serum in 1× PBST (0.1% Triton X-100 in PBS) for no more than 1 h at room temperature and then incubated with appropriately diluted primary antibodies at 4 °C overnight. The next day, the sections were washed three times in 1× PBS for 5 min each and then incubated with suitable secondary antibodies for 40 min to 1 h at room temperature in the dark. The sections were subsequently washed three times in 1× PBS for 5 min each and mounted with 50% glycerol. Finally, nail polish oil was applied to the four edges of the glass coverslip for further analysis. AlexaFluor-conjugated secondary antibodies were used to develop signals. Horseradish peroxidase-conjugated antibodies (Jackson ImmunoResearch) with a tyramine signal amplification kit (PerkinElmer) were used to amplify weak signals. Immunostaining images were acquired by an Olympus laser scanning confocal microscope (FV1200 and FV4000). ImageJ (NIH) and Photoline (21.00) software programs were used to analyze the images. Primary antibodies against the following were used for IF staining: F4/80 (Abcam, ab6640; 1:500), tdTomato (Rockland, 600-401-379; 1:1000 and Rockland, 200-101-379; 1:1000), SiglecF (R&D, AF1706; 1:300), PDGFRa (R&D, AF1062; 1:500), PDGFRb (eBioscience, 14-1402-82; 1:500), collagen I (Abcam, ab34710; 1:200), collagen IV (Millipore, ab769; 1:500), Ki67 (Abcam, ab15580; 1:200), SPC (Millipore, ab3786; 1:300), CD68 (Bio-Rad, MCA1957; 1:400), γ-H2AX (Thermo Fisher Scientific, IHC-00059; 1:200), CD11b (Thermo Fisher Scientific, 14-0112-82; 1:400), Ly6C (BD Biosciences, 553104; 1:200), and MerTK (eBioscience, 25-5751-82; 1:200). The following secondary antibodies were used: Alexa 488-conjugated donkey anti-rabbit IgG (Invitrogen, A21206; 1:1000), Alexa 555-conjugated donkey anti-rabbit IgG (Invitrogen, A31572; 1:1000), Alexa 647-conjugated donkey anti-rabbit IgG (Invitrogen, A31573; 1:1000), Alexa 488-conjugated donkey anti-rat IgG (Invitrogen, A21208; 1:1000), Alexa 647-conjugated donkey anti-rat IgG (Abcam, ab150155; 1:1000), Alexa 488-conjugated donkey anti-goat IgG (Invitrogen, A11055; 1:1000), Alexa 555-conjugated donkey anti-goat IgG (Invitrogen, A21432; 1:1000), Alexa 647-conjugated donkey anti-goat IgG (Invitrogen, A21447; 1:1000), ImmPRESS HRP-conjugated goat anti-rat IgG (Vector lab, MP-7444; 1:3) and HRP-conjugated donkey anti-rat IgG (Jackson ImmunoResearch, 712-035-153; 1:300).

### Sirius red staining

Sirius red staining was used to determine collagen deposition and indicate fibrotic areas in injury models, according to previously described protocols^59^. In detail, tissue sections were washed in 1× PBS for 15 min to remove OCT and fixed in 4% paraformaldehyde for 15 min at room temperature. The slides were then washed three times in 1× PBS for 5 min each and incubated in Bouin’s solution (5% acetic acid, 9% formaldehyde, and 0.9% picric acid) at room temperature overnight. On the next day, after being washed in running tap water until the yellow color disappeared, the slides were incubated in 0.1% Fast Green (Fisher, F-99) for 3 min and then were washed with tap water. The slides were then incubated in 0.1% Sirius red (Direct Red 80, Sigma, 0-03035) for 2 min. After being washed with double-distilled water, the slides were dehydrated with ethanol and xylene. Before microscopy, the slides were mounted with Neutral Balsam Mounting Medium (Sangon Biotech, E675007), and images were acquired with an Olympus microscope (BX53 and VS120).

### scRNA-seq by 10X Genomics

The isolated cell suspension was processed using the 10X Chromium system, with 13,000 cells being loaded during the generation of GEMs. Library preparation followed the guidelines provided in the Single-Cell 3′ Gene Expression Kit (v3.1) manual. Sequencing was conducted on an Illumina NovaSeq 6000 instrument using a PE150 kit.

### scRNA-seq data processing

Sequencing reads were aligned, annotated, and demultiplexed with CellRanger (v6.0.0) using the mm10-2020-A reference from 10X Genomics. Subsequent analyses were performed using the Seurat R package (v4.0.5)^62^. Quality control involved applying the subset function with thresholds of nFeature_RNA between 300 and 8000, nCount_RNA between 800 and 60,000, and mitochondrial gene expression below 15% to filter out low-quality cells. The doublet score was calculated by the Doublet-Detection package with default parameters^63^. Potential doublets were removed if the doublet score was greater than 40. PCA was conducted on the scaled expression data of the 3000 most variable genes selected by the “vst” method via the FindVariableFeatures function. Dimensionality reduction and clustering were then performed using the first 30 principal components. Clusters were defined using the markers mentioned above.

### RNA velocity and pseudotime analysis

To map the directions of differentiation trajectories, scVelo was employed to calculate the RNA velocity^64^. The previously filtered cells were utilized to perform UMAP analysis using the spliced assay data. The standard dynamical modeling workflow was then applied to generate the stream plot of velocities.

### DEGs and pathway enrichment analysis

A two-sided Wilcoxon rank-sum test was used to define marker genes for clusters and samples using the FindMarkers function in Seurat. *P* values were adjusted using the Benjamin–Hochberg FDR correction for the total number of comparisons. The gene enrichment analyses of the DEGs calculated above were performed using the DAVID webtool^65^. The cluster GSEA of Hallmark and KEGG pathways was performed by the *www_gsea()* function in the singleseqgset R package for monocytes, IMs, and AMs^66^.

### Macrophage phagocytosis

Mo-AMs and Mo-IMs were sorted by FACS, plated in 12-well plates at an appropriate density, and allowed to adhere for at least 2 h. Latex beads conjugated with FITC (Sigma, L1030; cell-to-bead ratio = 1:50) were washed three times with PBS and then incubated with fetal bovine serum (FBS) at 37 °C for 1 h at a ratio of 1 µL of beads to 100 µL of FBS. Concurrently, the cell culture medium was replaced with serum-free medium, and the cells were incubated for 1 h at 37 °C. After incubation, the beads were centrifuged at 10,656× *g* for 3 min and resuspended in serum-free medium. The beads were then equally distributed to each well and analyzed at specified time points using IF and FACS.

### Macrophage polarization

Mo-AMs and Mo-IMs were sorted by FACS and cultured in 12-well plates at 37 °C under 5% CO₂ for 3 h. M1 or M2 macrophage polarization was induced by stimulation with IFN-γ (50 ng/mL, PeproTech, #315-05) plus LPS (100 ng/mL, Solarbio, #L8880) or IL-4 (20 ng/mL, Solarbio, #P00021), respectively. After 24 h of culture, the cells were harvested for RNA extraction, and qRT-PCR was performed to assess the expression levels of polarization-related genes.

### RNA extraction

The lungs were collected for total RNA isolation using TRIzol reagent (Invitrogen, 15596018). In detail, 1 mL of TRIzol reagent was added to dissolve each sample and incubated for 5 min at room temperature (RT). The samples were subsequently centrifuged at 10,656× *g* for 5 min at 4 °C, after which the supernatant was transferred to a new 1.5-mL Eppendorf tube, followed by the addition of 200 μL of chloroform. Then the samples were intensely vortexed for 15–20 s and set aside for 15 min at RT. Next, the samples were centrifuged at 4 °C at 10,656× *g* for 15 min, after which the supernatant was transferred to another new 1.5-mL Eppendorf tube. Afterward, 500 μL of isopropyl alcohol was added to the samples, which were mixed thoroughly and set aside at RT for 10 min. The samples were then centrifuged at 10,656× *g* for 15 min at 4 °C and washed twice with 75% EtOH. Finally, after the precipitate was air-dried, appropriate amounts of RNase/DNase-free water were added to dissolve the RNA.

### qRT-PCR

One microgram of total RNA was reverse transcribed into cDNA using the TaKaRa PrimeScript^TM^ RT reagent Kit with gDNA Eraser (Perfect Real Time, Takara, RP074A). Two separate steps were needed: first, 42 °C for 2 min for the genomic DNA elimination reaction; second, 37 °C for 15 min, 85 °C for 5 s, and hold at 4 °C in a PCR machine for reverse transcription. The synthesized cDNA was diluted to 10 ng/μL with double-distilled water (ddH_2_O) and then stored at −20 °C for further experiments. qRT-PCR was carried out using a QuantStudio^TM^ 6 Flex Real-Time PCR System (Thermo Fisher Scientific) with Invitrogen SYBR Green qRT-PCR reagents (Invitrogen, 4367659). A 10-μL reaction system was constructed that included 5 μL of 2× SYBR Green, 1 μL of forward and reverse mixed primers, 1 μL of diluted cDNA template, and 3 μL of ddH_2_O. The qRT-PCR program was as follows: 95 °C for 10 min, 40 repetitions of 95 °C for 5 s and 60 °C for 30 s. The results were normalized to those of GAPDH, with the control set as 1. All the primers used are listed in Supplementary Table S1.

### Statistical analysis

Data are expressed as the mean ± SEM of biological replicates. Unpaired Student’s *t*-tests were conducted for single-variable comparisons between two groups using GraphPad Prism 7. For assessments involving two independent variables (treatment and time), two-way ANOVA was employed, followed by Tukey’s HSD for post hoc multiple comparisons. Bonferroni correction was applied to adjust the significance levels to control for type I error risk. A *P* value adjusted to less than 0.05 was deemed significant. All experiments and analyses were performed in a blinded manner by separate researchers.

## Supplementary information

Supplementary Information

## Data Availability

All the data that support the findings of this study are provided within the paper and its supplementary information. scRNA-seq data that support the findings of this study are deposited in the Gene Expression Omnibus under accession code GSE279529, which can be accessed at https://www.ncbi.nlm.nih.gov/geo/query/acc.cgi?acc=GSE279529 using the token “mdgxyemmjtqtdiv”.
