# Supplementary material for: Lineage tracing reveals the origins and dynamics of macrophages in lung injury and repair
Source: Cell Discov. 2026 Jan 14;12:3. doi: 10.1038/s41421-025-00859-0 (PMC12804754; doi:10.1038/s41421-025-00859-0)
Supplement: Supplementary file 1 — Supplementary Information [file 41421_2025_859_MOESM1_ESM.pdf]

Supplementary Information for

Lineage tracing reveals the origins and dynamics of macrophages in lung injury and repair

Hengwei Jin*, Jialing Mou, Huan Zhu, Kuo Liu, Mingjun Zhang, Zhenqian Zhang, Stefan Pflanz, Karim EI Kasmi, Zhaoyuan Liu, Florent Ginhoux, Kathy O. Lui*, Bin Zhou*

*Correspondence to: jinhw@sibcb.ac.cn (H.J.), kathyolui@cuhk.edu.hk (K.O.L.), zhoubin@sibs.ac.cn (B.Z.)

The following sections include:

Supplementary Figures S1 to S16

Supplementary Table S1

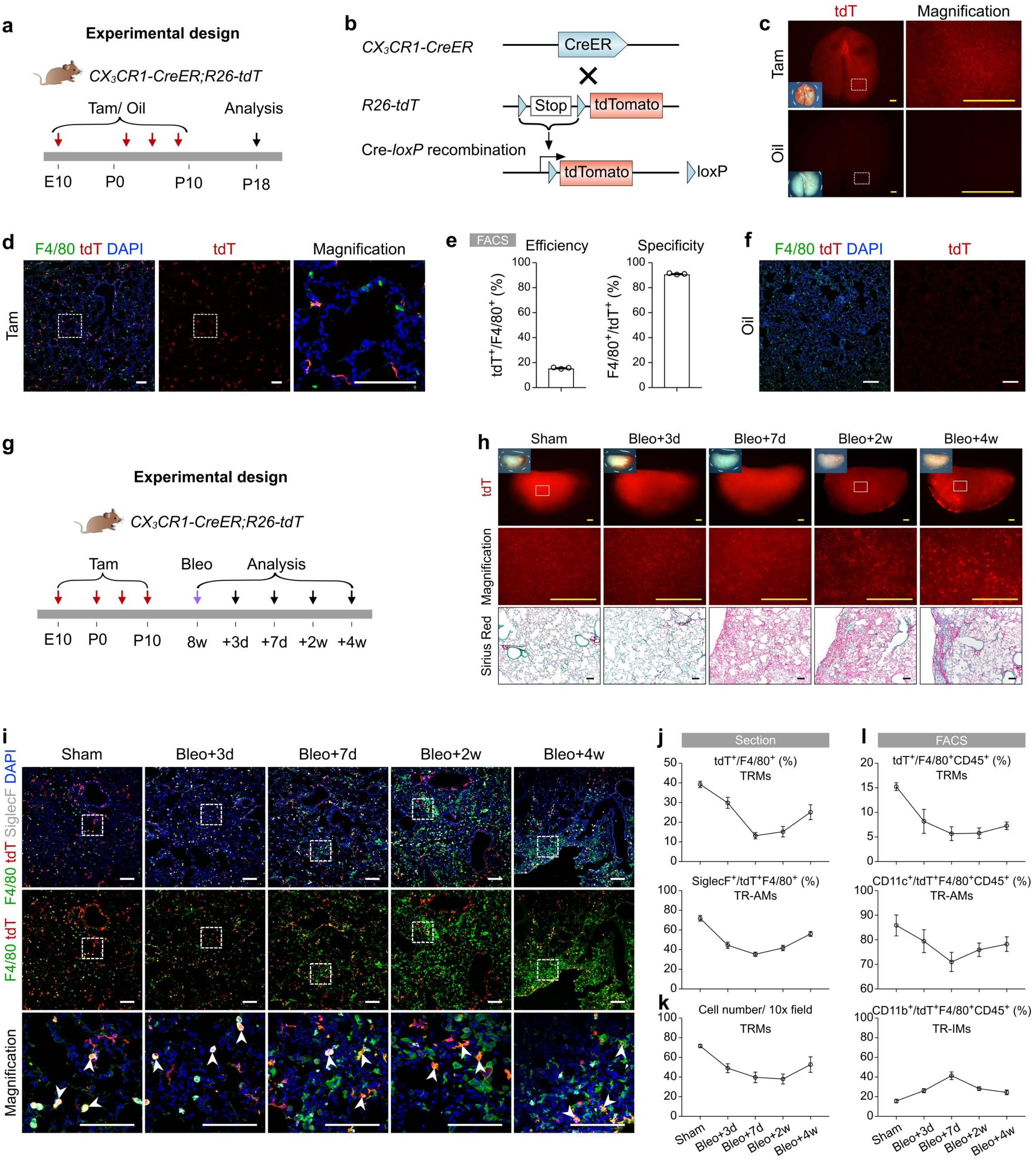

Supplementary Fig. S1. Tissue resident macrophages are lost in response to bleomycin-induced lung injury. a Schematic showing experimental design. Tam, tamoxifen. **b** Genetic lineage tracing strategy by Cre-*loxP* recombination in CX3CR1$^+$ cells after tam treatment. **c** Whole-mount bright field and epifluorescence images of lungs. Boxed region is magnified. **d** Immunostaining for tdT and F4/80 on tissue sections of lungs with tam. Boxed region is magnified. **e** FACS analysis of the percentage of F4/80$^+$ macrophages expressing tdT (efficiency), and the percentage of tdT$^+$ cells expressing F4/80 (specificity) from lungs. Data are the mean ± SEM; n=3 mice per group. **f** Immunostaining for tdT and F4/80 on tissue sections of lungs with oil. **g** Schematic showing experimental design. Bleo, bleomycin. **h** Whole-mount bright field, epifluorescence, and sirius red images of lungs after bleomycin treatment. Boxed region is magnified. **i** Immunostaining for tdT, F4/80 and SiglecF on tissue sections of lungs. Boxed region is magnified. White arrowheads indicating TRMs. **j** Quantitative analysis of the percentage of F4/80$^+$ macrophages expressing tdT, and the percentage of alveolar macrophages (SiglecF$^+$) in labeled tdT$^+$ macrophages from lung immunostaining sections. Data are the mean ± SEM; n=3~5 mice per group. **k** Quantification analysis of the cell number of TRMs (tdT$^+$F4/80$^+$) in each 10x field. Data are the mean ± SEM; n=3~5 mice per group. **l** Flow cytometric and quantification analyses of the percentage of tdT$^+$ cells in CD45$^+$F4/80$^+$ macrophages (TRMs), the percentage of CD11c$^+$ alveolar macrophages in tdT$^+$ macrophages (TR-AMs), and the percentage of CD11b$^+$ interstitial macrophages in tdT$^+$ macrophages (TR-IMs) from lungs. Data are the mean ± SEM; n=3~5 mice per group. TRMs, tissue resident macrophages; TR-AMs, tissue resident alveolar macrophages; TR-IMs, tissue resident interstitial macrophages. Scale bars, yellow, 1mm; white and black, 100 μm. Each image is representative of 3–5 individual samples.

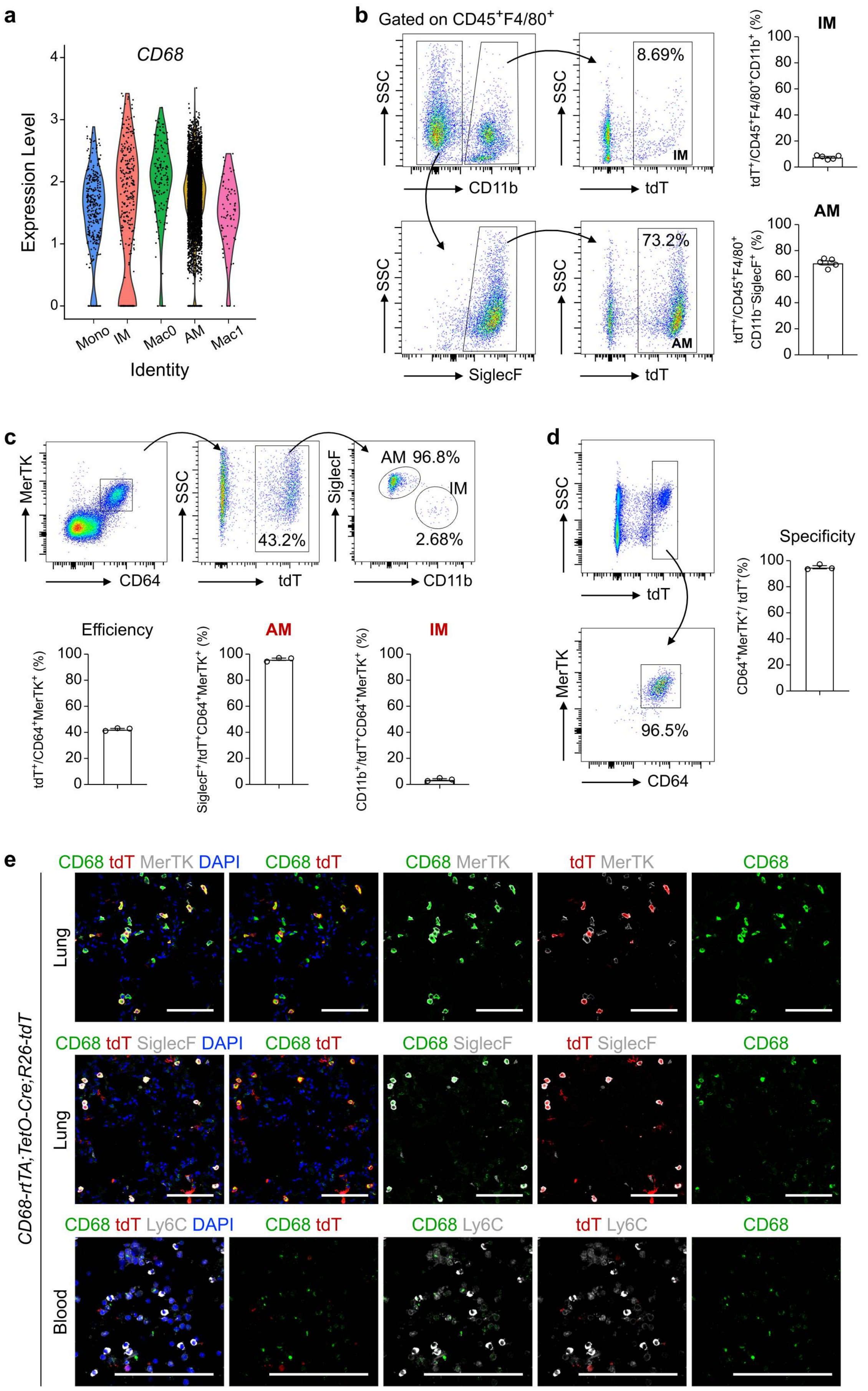

Supplementary Fig. S2. *CD68-rtTA* specifically targets lung alveolar macrophages. a Violin plots showing the expression levels of *CD68* in monocytes (mono), interstitial macrophages (IM), Mac0, alveolar macrophages (AMs), and Mac1. **b** Flow cytometric and quantification analyses of the percentage of tdT^+ cells among lung IMs ($CD45^+F4/80^+CD11b^+$) and AMs ($CD45^+F4/80^+CD11b^-SiglecF^+$) in *CD68-rtTA;TetO-Cre;R26-tdT* mice. Data are the mean ± SEM; n=5 mice per group. **c** Flow cytometric and quantification analyses of the percentage of tdT^+ cells in $CD64^+MerTK^+$ lung macrophages, and the percentage of $SiglecF^+$ AMs and $CD11b^+$ IMs within the tdT^+ macrophages in *CD68-rtTA;TetO-Cre;R26-tdT* mice. Data are the mean ± SEM; n=3 mice per group. **d** Flow cytometric and quantification analyses of the percentage of $CD64^+MerTK^+$ lung macrophages within tdT^+ cells in *CD68-rtTA;TetO-Cre;R26-tdT* mice. Data are the mean ± SEM; n=3 mice per group. **e** Immunostaining for CD68, tdT, MerTK, SiglecF, and Ly6C on lung tissue and blood samples from *CD68-rtTA;tetO-Cre;R26-tdT* mice. Scale bars, white, 100 μm. Each image is representative of 3–5 individual samples.

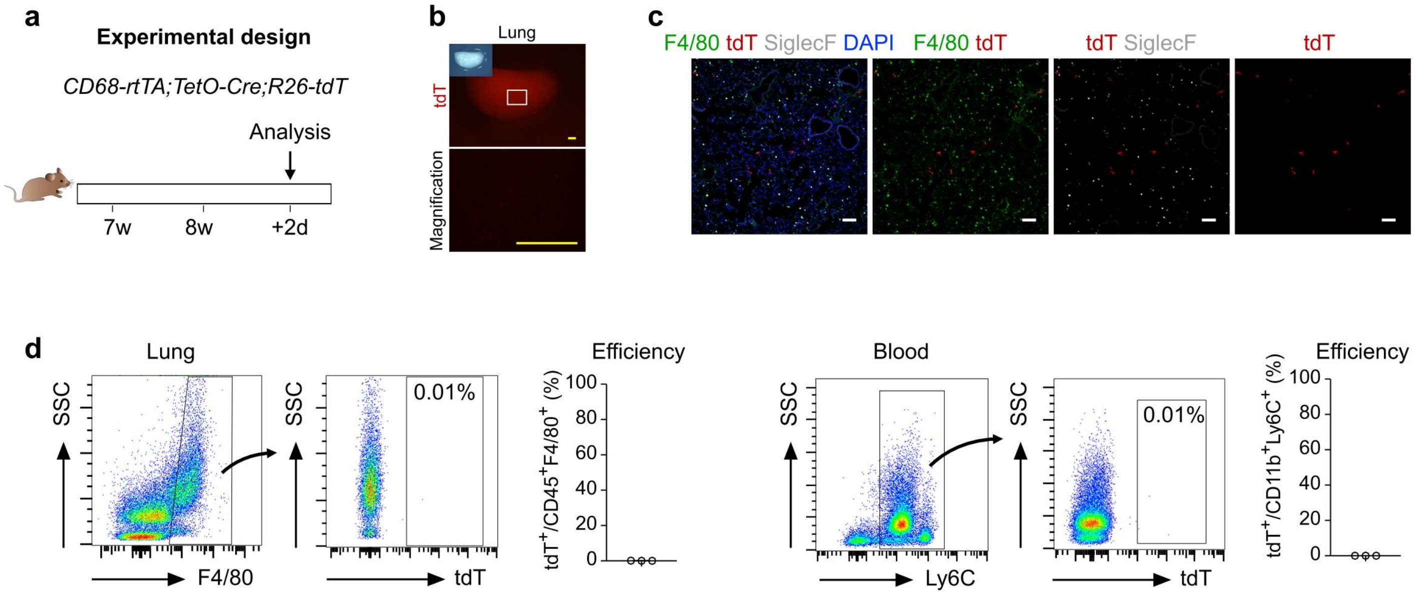

Supplementary Fig. S3. *CD68-rtTA* barely targets lung macrophages without Dox administration. a Schematic showing experimental design. **b** Bhole-mount bright field and epifluorescence images of lungs. Boxed region is magnified. **c** Immunostaining for tdT, F4/80 and SiglecF on tissue sections of (b). **d** Flow cytometric and quantification analyses of the percentage of tdT$^+$ cells in CD45+F4/80+ macrophages from lungs (left), and the percentage of tdT$^+$ cells in CD45$^+$Ly6C$^+$ monocytes from blood (right). Data are the mean ± SEM; n=3 mice per group. Scale bars, yellow, 1mm; white, 100 μm. Each image is representative of 3–5 individual samples.

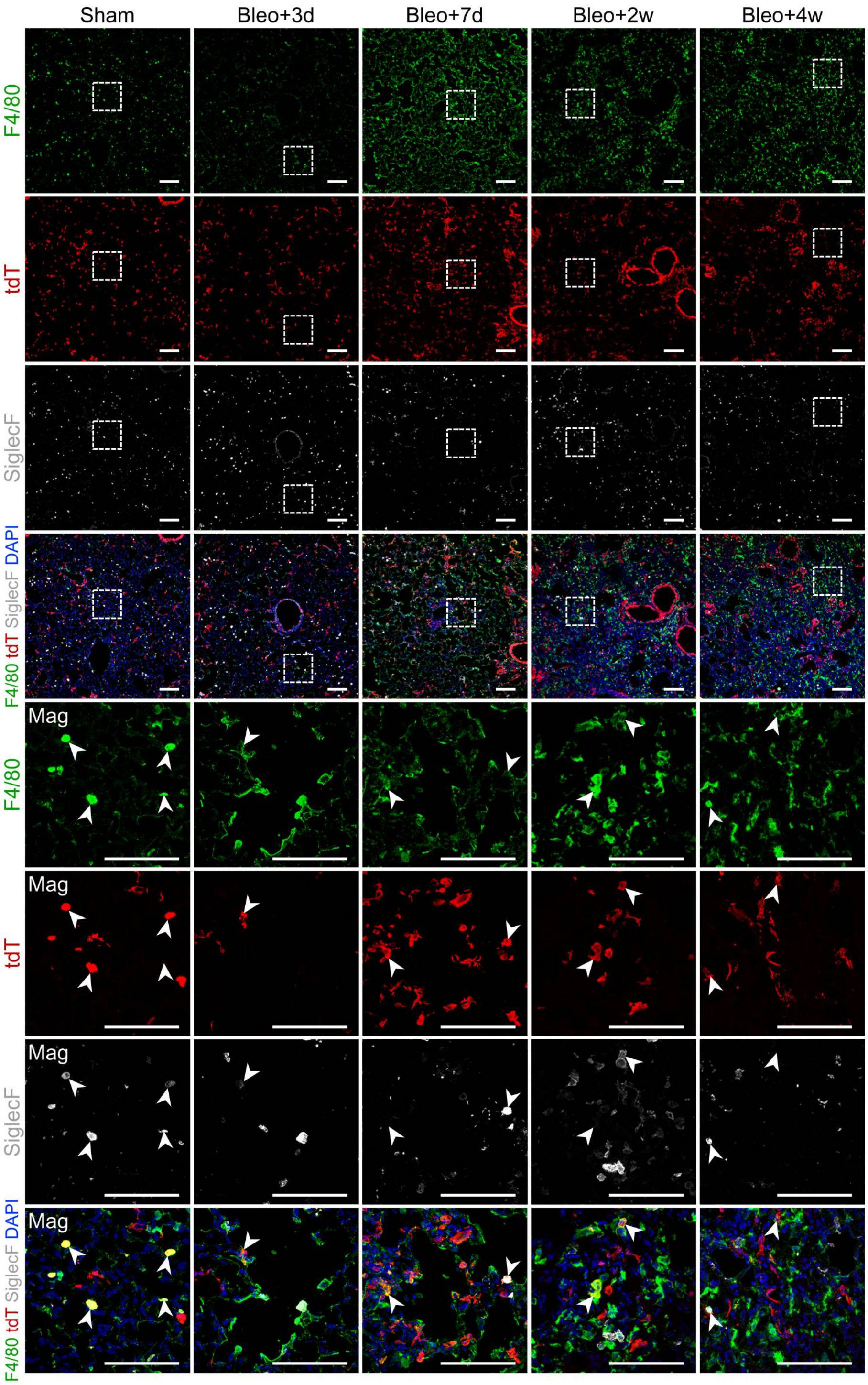

Supplementary Fig. S4. Decomposition of fluorescence channels from Fig. 2c. Scale bars, white, 100 μm.

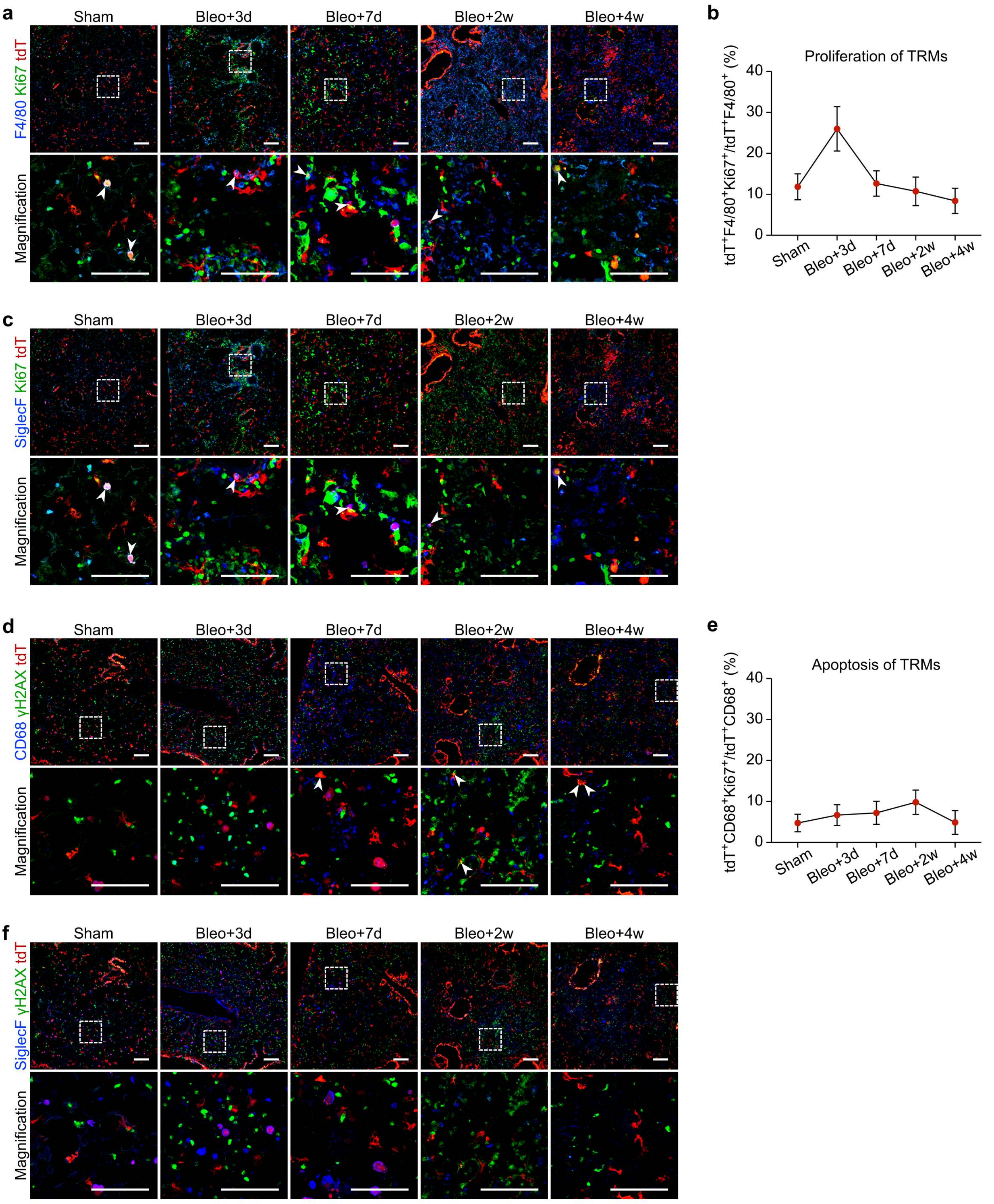

Supplementary Fig. S5. Bleomycin induces proliferation of tissue resident macrophages in the lungs. a, c Immunostaining for tdT, Ki67, and F4/80 (a) or SiglecF (c) on tissue sections of lungs. Boxed region is magnified. White arrowheads indicating proliferated TRMs in (a) or TR-AMs in (c), respectively. **b** Quantification analysis of the percentage of the TRMs proliferation ($F4/80^+tdT^+Ki67^+$ cells) in TRMs ($F4/80^+tdT^+$ cells) in each 10x filed. Data are the mean ± SEM; n=3~5 mice per group. **d, f** Immunostaining for tdT, γH2AX, and CD68 (d) or SiglecF (f) on tissue sections of lungs. White arrowheads indicating apoptotic TRMs in (d) or TR-AMs in (f), respectively. Boxed region is magnified. **e** Quantification analysis of the percentage of TRMs apoptosis ($CD68^+tdT^+γH2AX^+$ cells) in TRMs ($CD68^+tdT^+$) in each 10x filed. Data are the mean ± SEM; n=3~5 mice per group. Scale bars, 100 μm. Each image is representative of 3–5 individual samples.

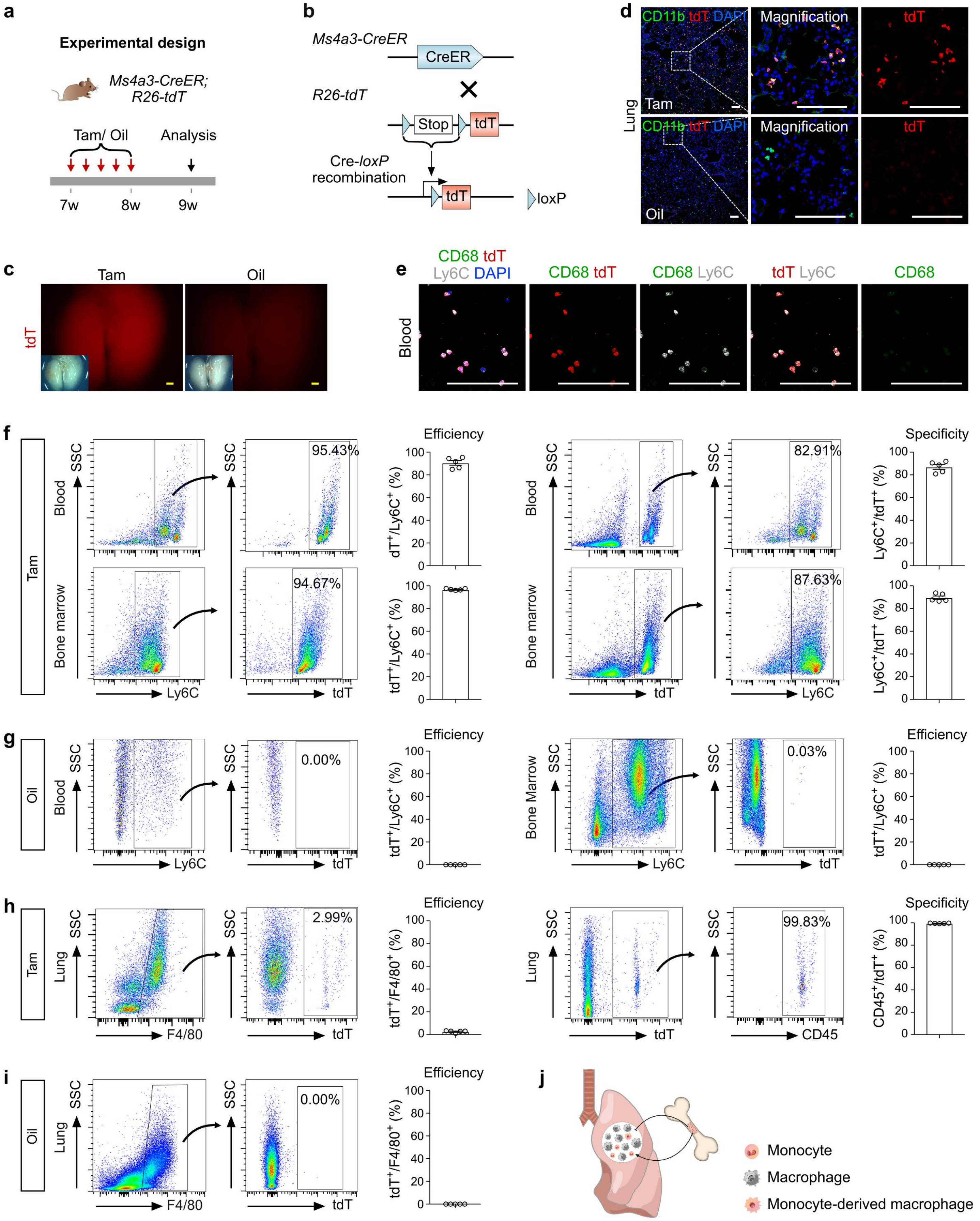

Supplementary Fig. S6. Characterization of *Mas4a3-CreER* mouse line. **a** Schematic showing experimental design. Tam, tamoxifen. **b** Genetic lineage tracing strategy by Cre-*loxP* recombination in Ms4a3⁺ cells after tam treatment. **c** Whole-mount bright field and epifluorescence images of lungs after tam or oil treatment. **d** Immunostaining for tdT and CD11b on tissue sections of lungs. Boxed region is magnified. **e** Immunostaining for CD68, tdT and Ly6C on blood samples. **f** Flow cytometric and quantification analysis of the percentage of tdT⁺ cells in CD45⁺Ly6C⁺ monocytes (efficiency) and the percentage of tdT⁺ cells expressing Ly6C (specificity) from blood and bone marrow after tam. Data are the mean ± SEM; n=5 mice per group. **g** Flow cytometric and quantification analysis of the percentage of tdT⁺ cells in CD45⁺Ly6C⁺ monocytes from blood and bone marrow after oil. Data are the mean ± SEM; n=5 mice per group. **h** Flow cytometric and quantification analyses of the percentage of tdT⁺ cells in CD45⁺F4/80⁺ macrophages (efficiency) and the percentage of tdT⁺ cells expressing CD45 (specificity) from lung after tam. Data are the mean ± SEM; n=5 mice per group. **i** Flow cytometric and quantification analyses of the percentage of tdT⁺ cells in CD45⁺F4/80⁺ macrophages from lung after oil. Data are the mean ± SEM; n=5 mice per group. **j** Cartoon showing the labeling results after recombination by tamoxifen. Scale bars, yellow, 1 mm; white, 100 μm. Each image is representative of 3–5 individual samples.

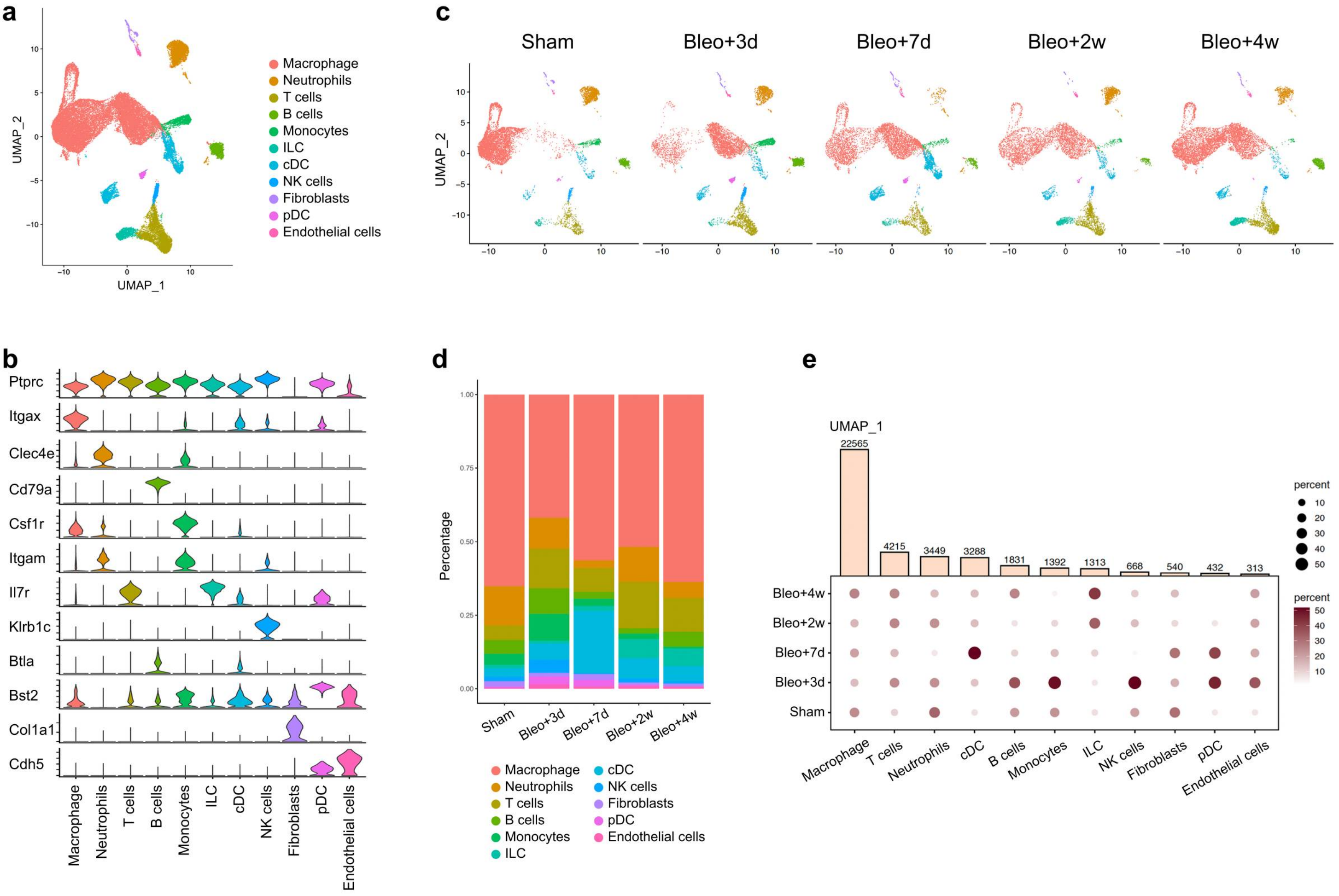

Supplementary Fig. S7. Single-cell analysis of lung immune cells. a Uniform Manifold Approximation and Projection (UMAP) embedding showing cells colored by cell type identity. **b** Violin plots of cluster-defining genes. **c** The colored dots on the UMAP illustrating densities and distribution of cells at individual time points after bleomycin-induced injury. **d** Bar plot of percentage of cluster contributing per time point. **e** Dot plot showing the percentage of each cluster contributing at different time points. The total percentage of one cluster is 100%.

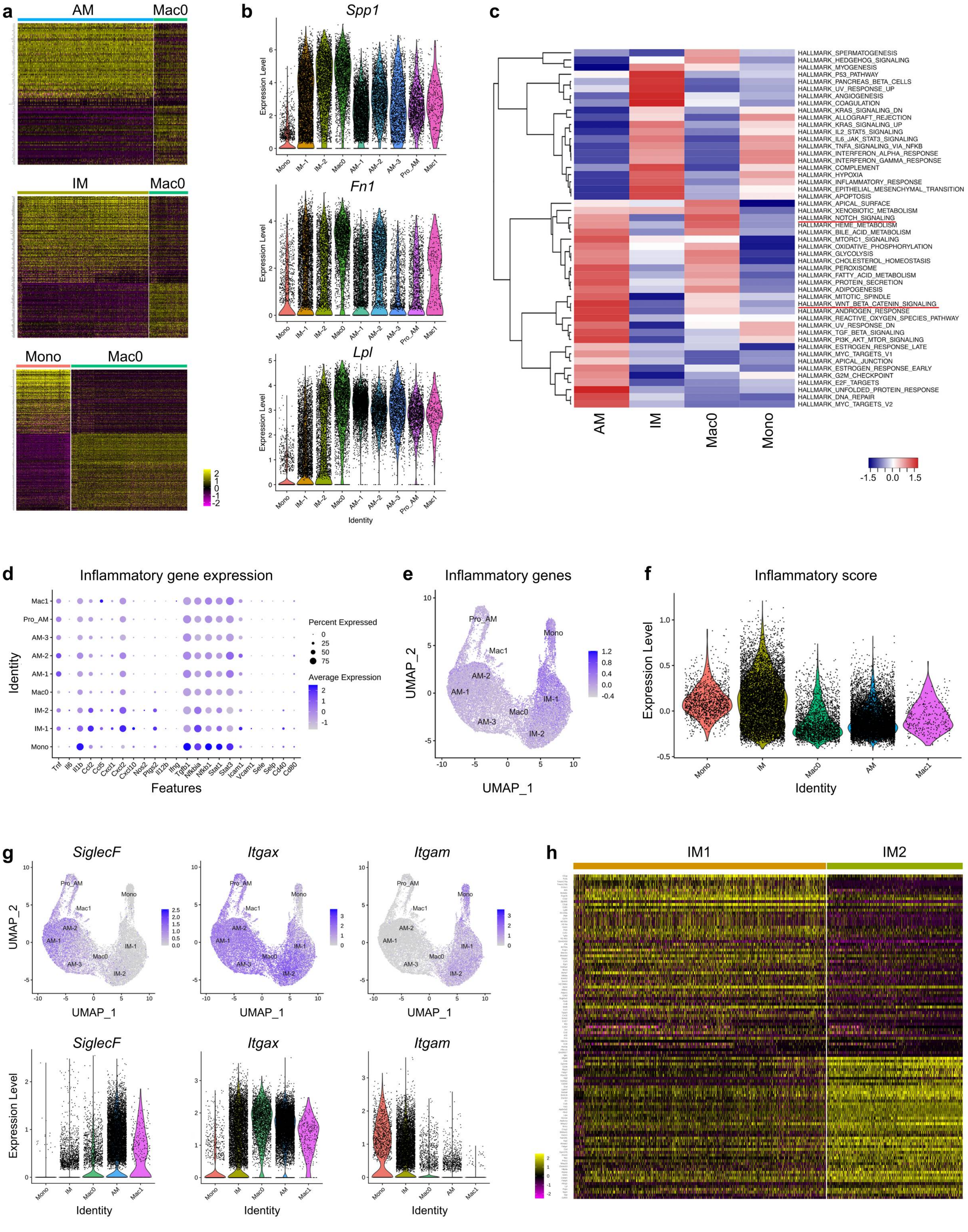

Supplementary Fig. S8. Single-cell RNA-seq analysis of myeloid cell populations. **a** Heatmap showing the DEGs of Mac0 versus other clusters. **b** Violin plots illustrating the highly expressed genes in the cluster of Mac0. **c** Heatmap displaying the representative differentially enriched signaling pathways between AM, IM, Mac0, and monocyte. **d** Dot plot displaying the expression levels of key inflammatory genes in the Mac0 cluster. **e, f** FeaturePlot and Violin plots showing an overall inflammatory score along the differentiation trajectory. **g** FeaturePlot and Violin plots showing the expression of *SiglecF*, *Itgax*, and *Itgam*. **h** Heatmap showing the DEGs between the IM1 and IM2 clusters.

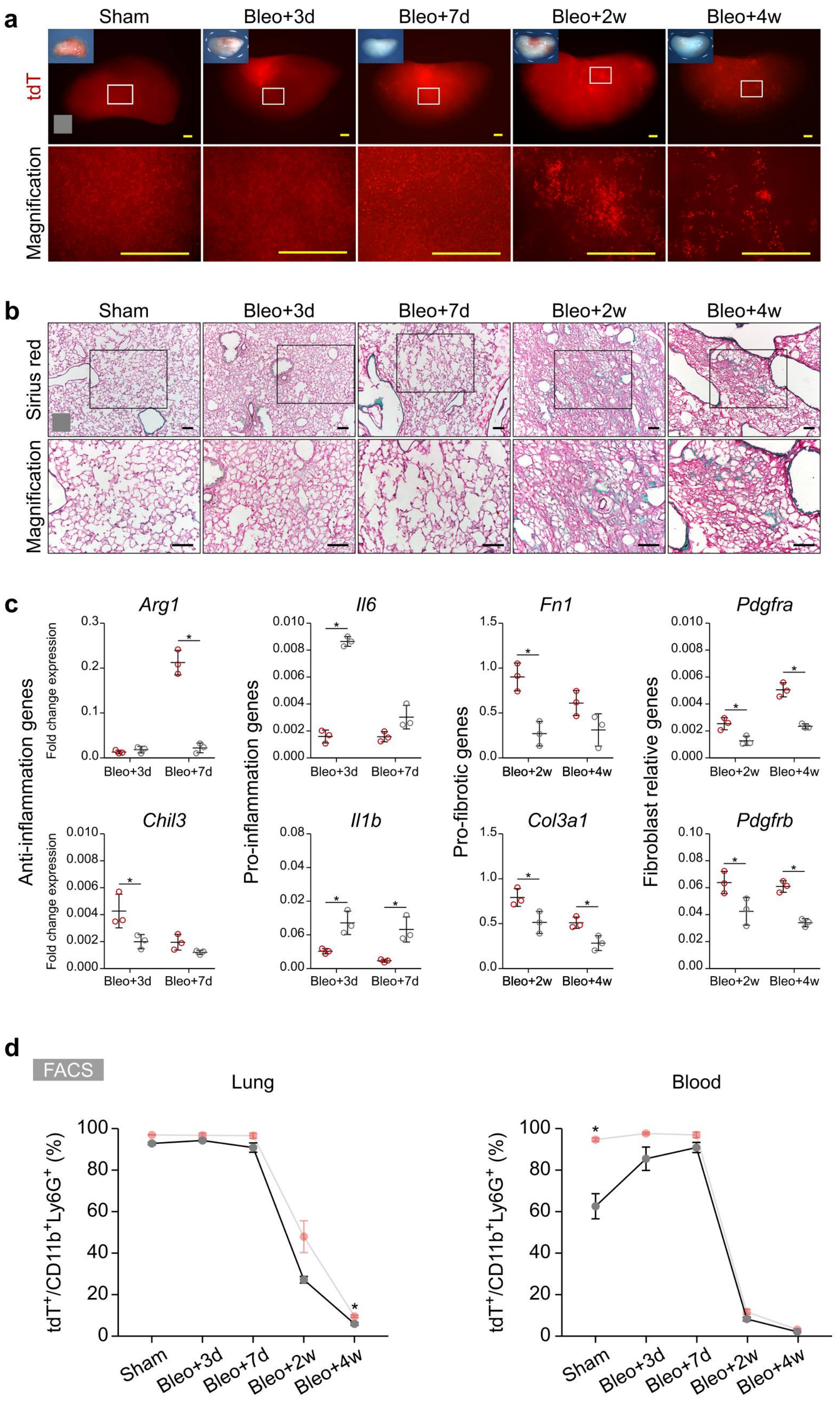

Supplementary Fig. S9. Histological and cytological analyses after ablation monocytes. a Whole-mount bright field and epifluorescence images of *Ms4a3-CreER;R26-tdT/iDTR* lungs after bleomycin treatment. Boxed region is magnified. **b** Sirius red staining of the *Ms4a3-CreER;R26-tdT/iDTR* lung tissue sections treated with bleomycin. Boxed region is magnified. **c** Gene mRNA expression in bleomycin-treated lungs at different stages. Data are the mean ± SEM; n=3 mice per group. **d** Flow cytometric and quantification analysis of the percentage of tdT^+ cells in $CD45^+CD11b^+Ly6G^+$ neutrophils from lung tissue and blood samples. Data are the mean ± SEM; n=5 mice per group. Scale bars, yellow, 1mm; black, 100 μm. Each image is representative of 5 individual samples.

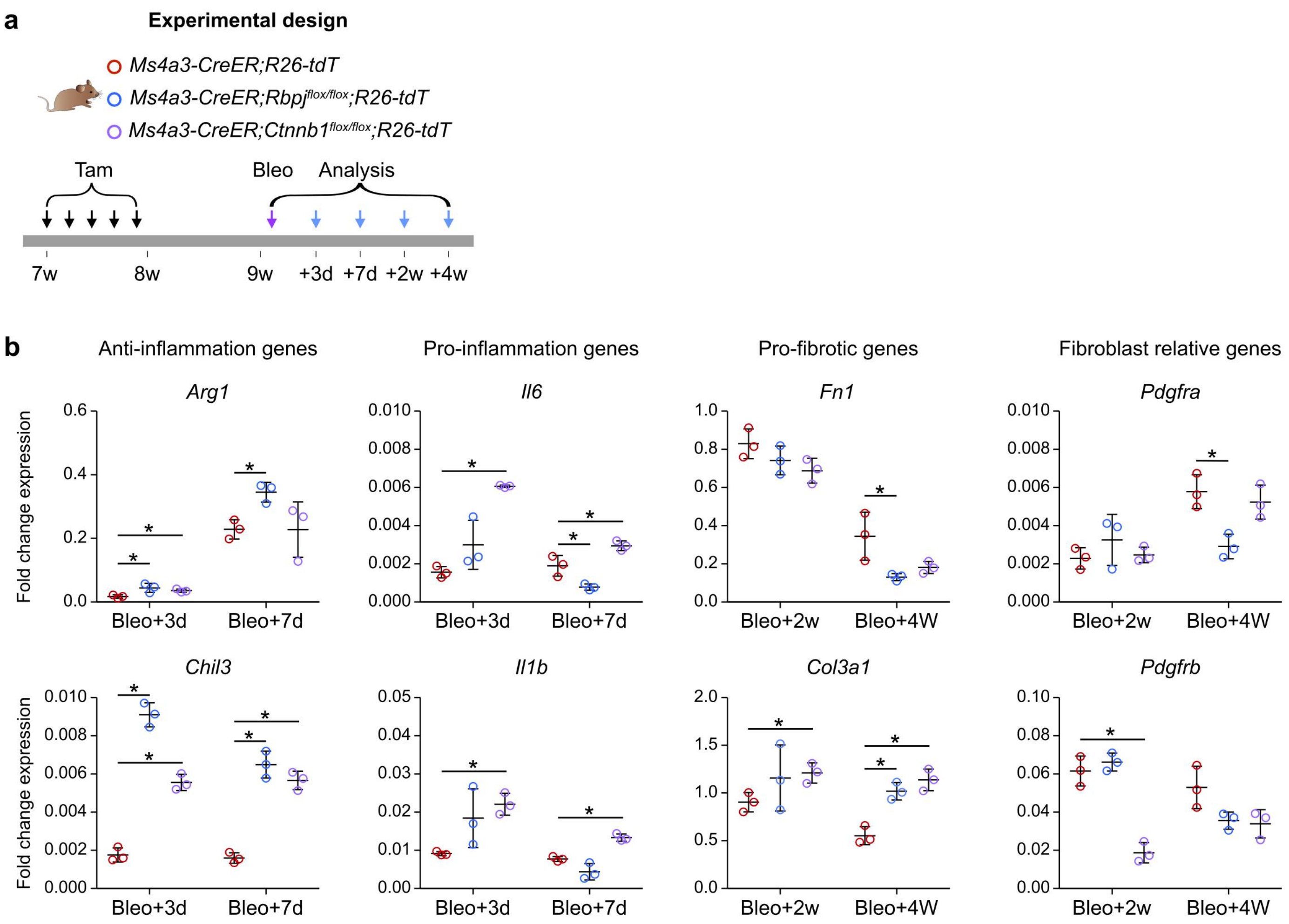

Supplementary Fig. S10. Quantitative real-time PCR analysis. a Schematic showing experimental design. Tam, tamoxifen; Bleo, bleomycin. **b** qRT-PCR analysis detected the mRNA expression levels of inflammatory and fibrotic genes in different mice groups after bleomycin-induced injury. Data are the mean ± SEM; n=3 mice per group.

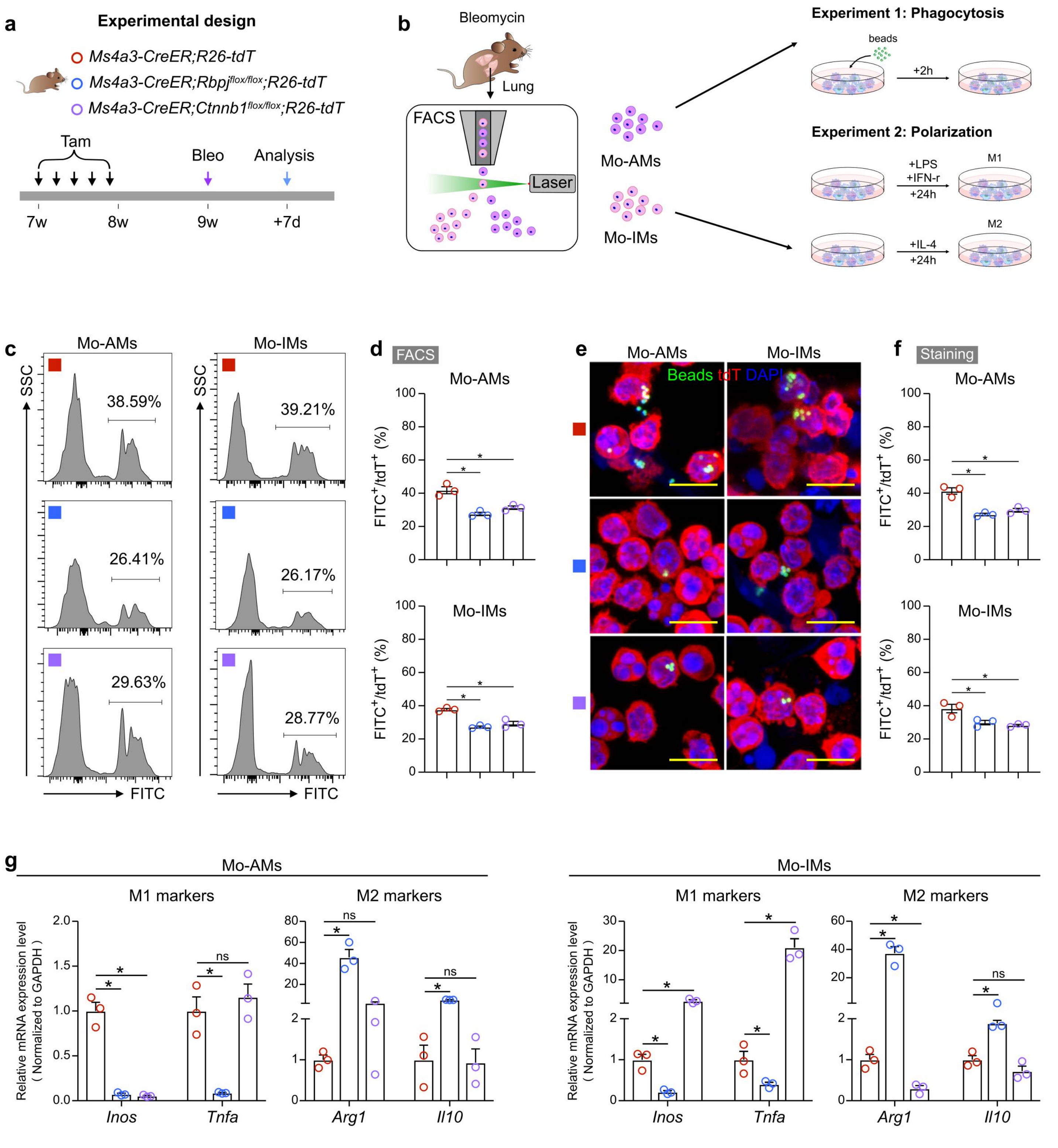

Supplementary Fig. S11. Conditional konckout *Rbpj* or *Ctnnb1* in monocytes impairs the function of Mo-AMs and Mo-IMs. a Schematic showing experimental design. Tam, tamoxifen; Bleo, bleomycin. **b** Schematic illustration of the experiment workflow. **c, d** Flow cytometric and quantification analysis of tdT⁺ Mo-AMs and Mo-IMs express FITC. Data are the mean ± SEM; n=3 mice per group. **e** Immunostaining of tdT⁺ Mo-AMs and Mo-IMs treated with FBS-coated FITC beads. **f** Quantification analysis of the percentage of FITC⁺ cells among tdT⁺ Mo-AMs and Mo-IMs. Data are the mean ± SEM; n = 3 mice per group. **g** mRNA expression levels of M1/M2 polarization genes in tdT⁺ Mo-AMs and Mo-IMs. Data are the mean ± SEM; n = 3 mice per group. ns, non-significant. Scale bars, 1 mm. Each image is representative of 3 individual samples.

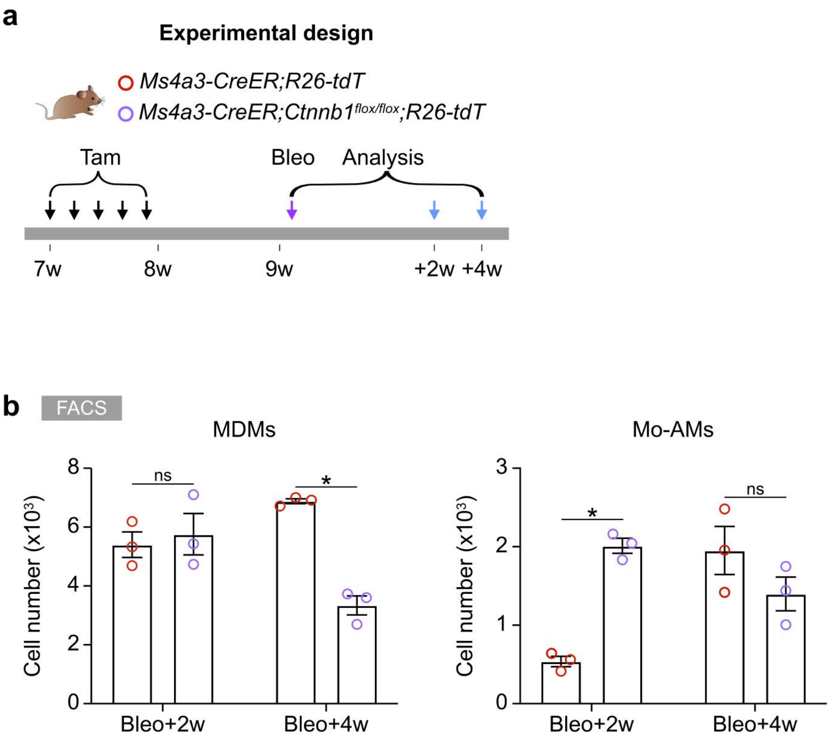

Supplementary Fig. S12. Absolute cell numbers of MDMs and Mo-AMs in *Ctnnb1* knockout mice after Bleomycin. a Schematic showing experimental design. Tam, tamoxifen; Bleo, bleomycin. **b** Quantification of absolute cell numbers of MDMs and Mo-AMs by FACS. Data are the mean ± SEM; n = 3 mice per group. ns, non-significant.

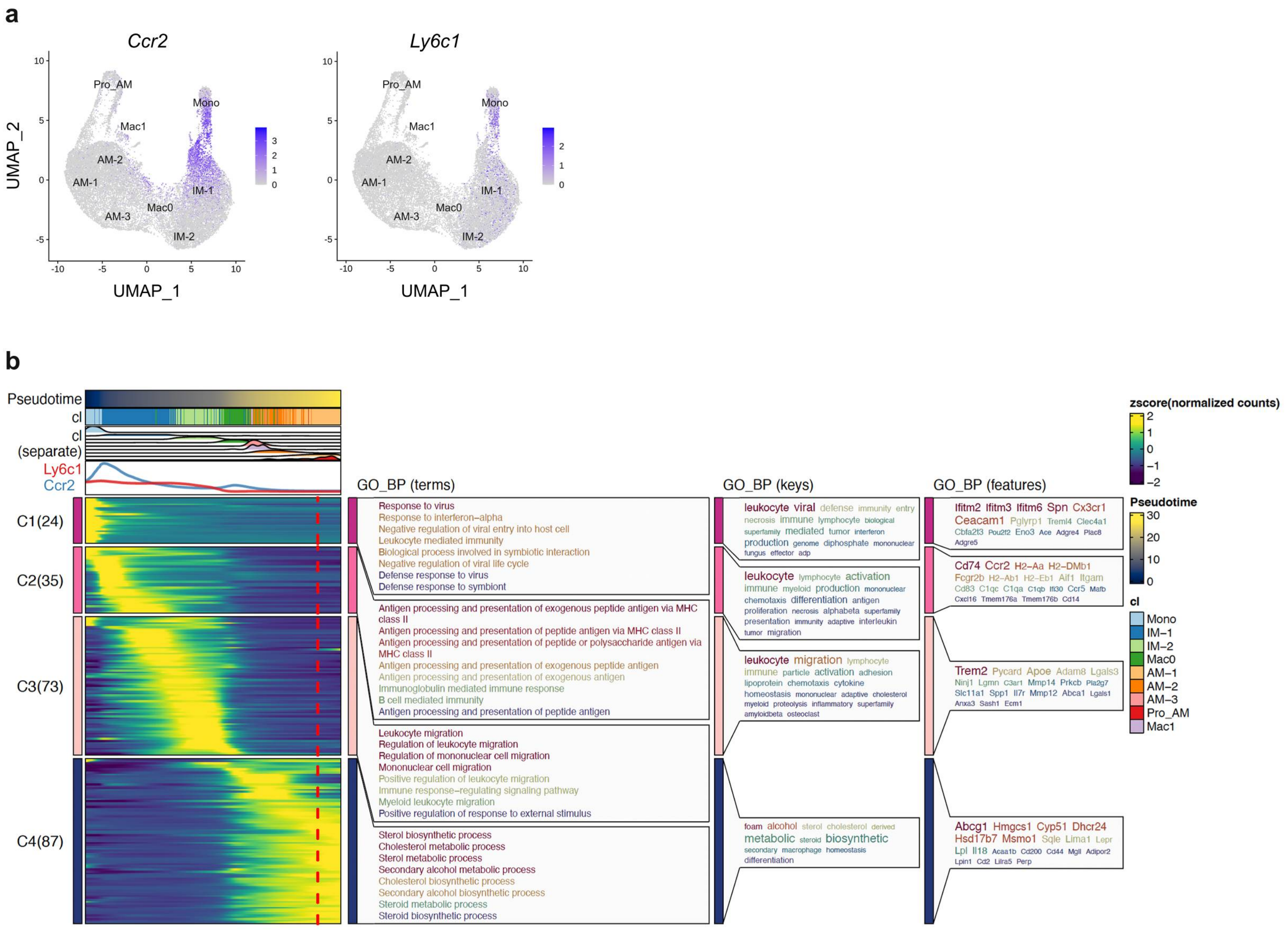

Supplementary Fig. S13. CCR2⁺Ly6C^high inflammatory monocytes serve as the primary source of IMs. a FeaturePlot showing the expression level of *Ccr2* and *Ly6c1* along the differentiation trajectory. **b** Pseudotime trajectory analysis of macrophage-related cell populations.

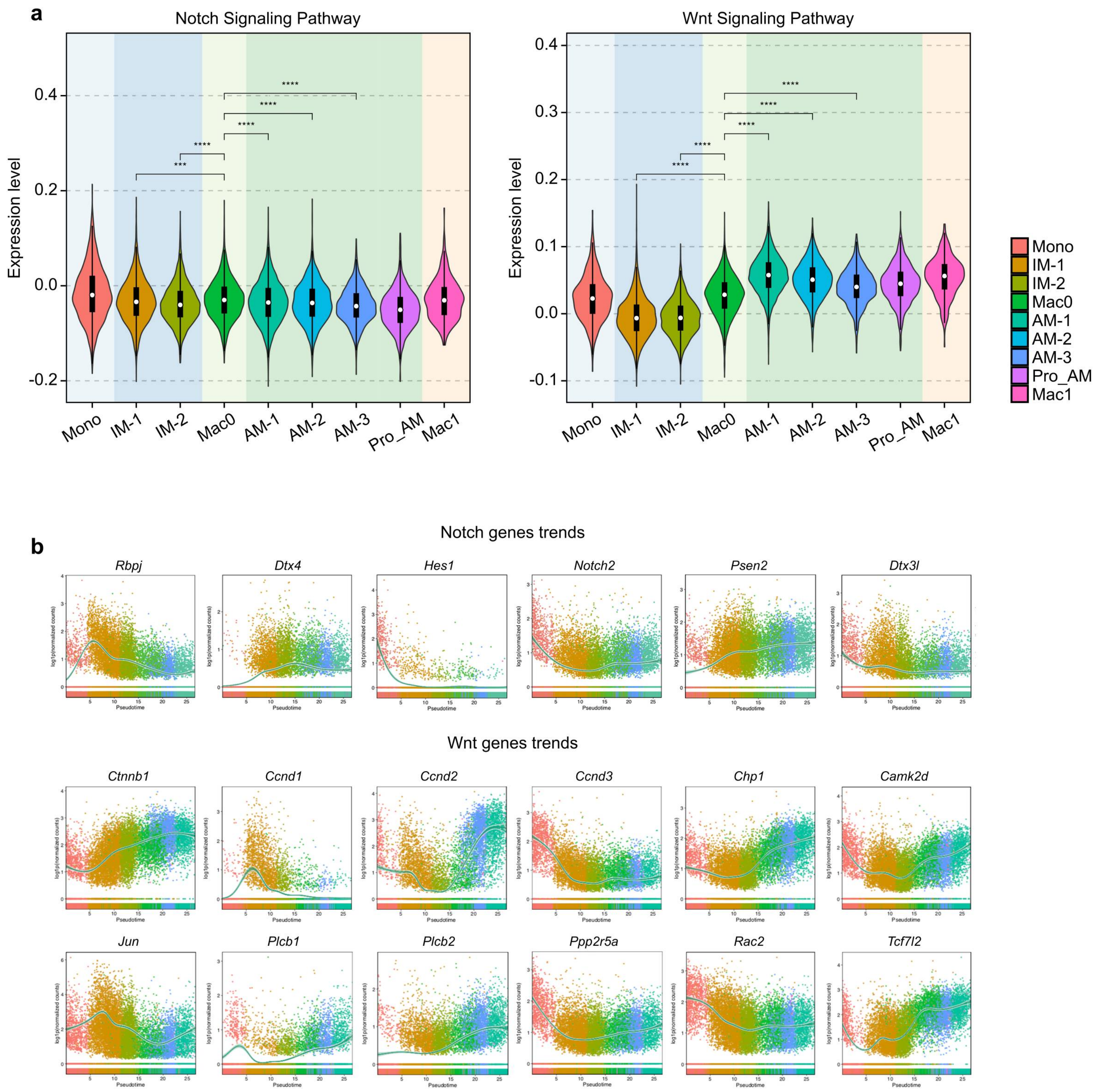

Supplementary Fig. S14. Crosstalk between Notch and Wnt signaling pathways in regulating monocyte to AM differentiation. a Activity scores of the Notch and Wnt signaling pathways along the monocyte differentiation trajectory. **b** Expression trend of key genes in Notch and Wnt signaling pathway.

a Graphical Abstract

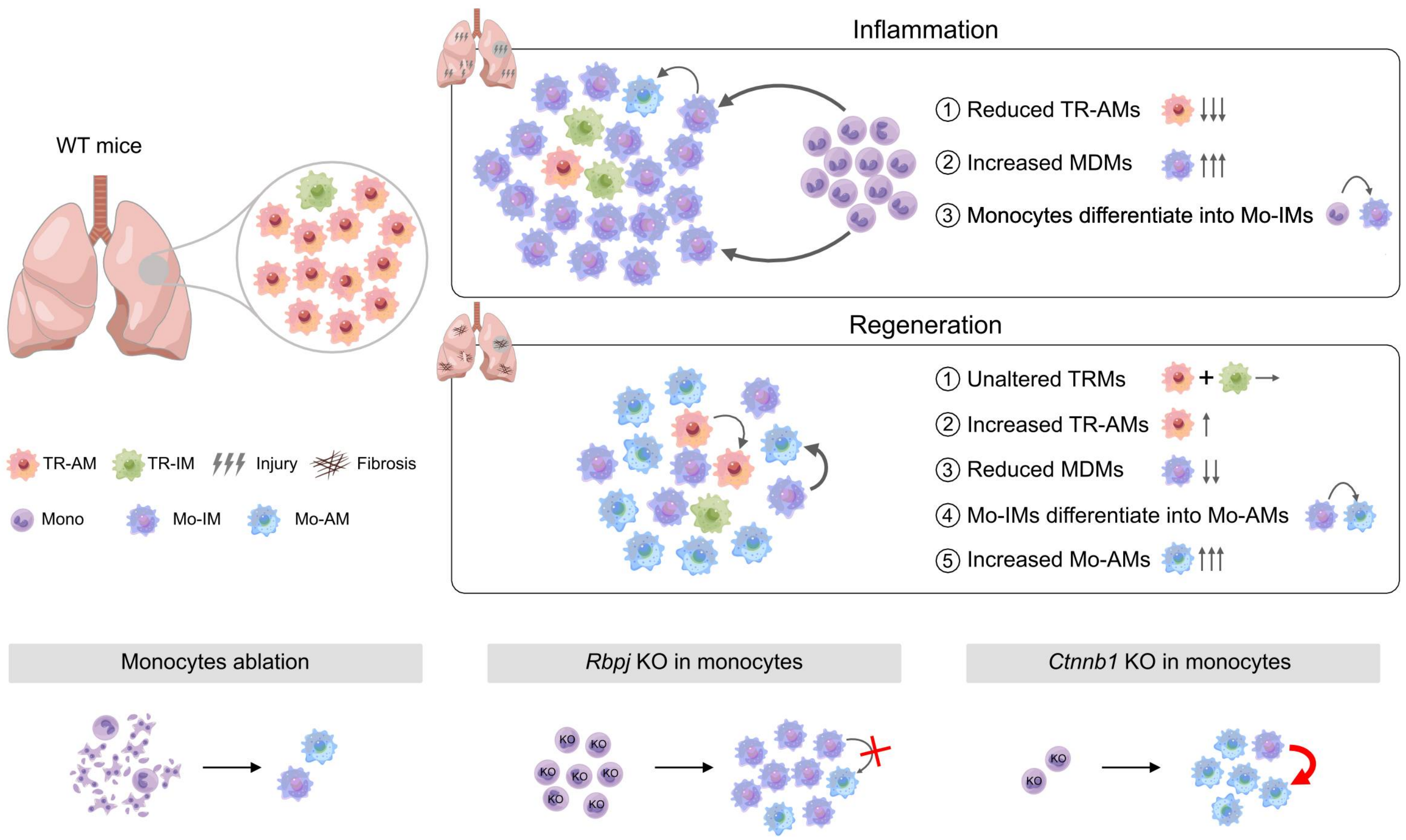

Supplementary Fig. S15. Graphical abstract of the study. a Graphical abstract depicting the dynamic alterations of TRMs and MDMs throughout the processes of lung injury and repair.

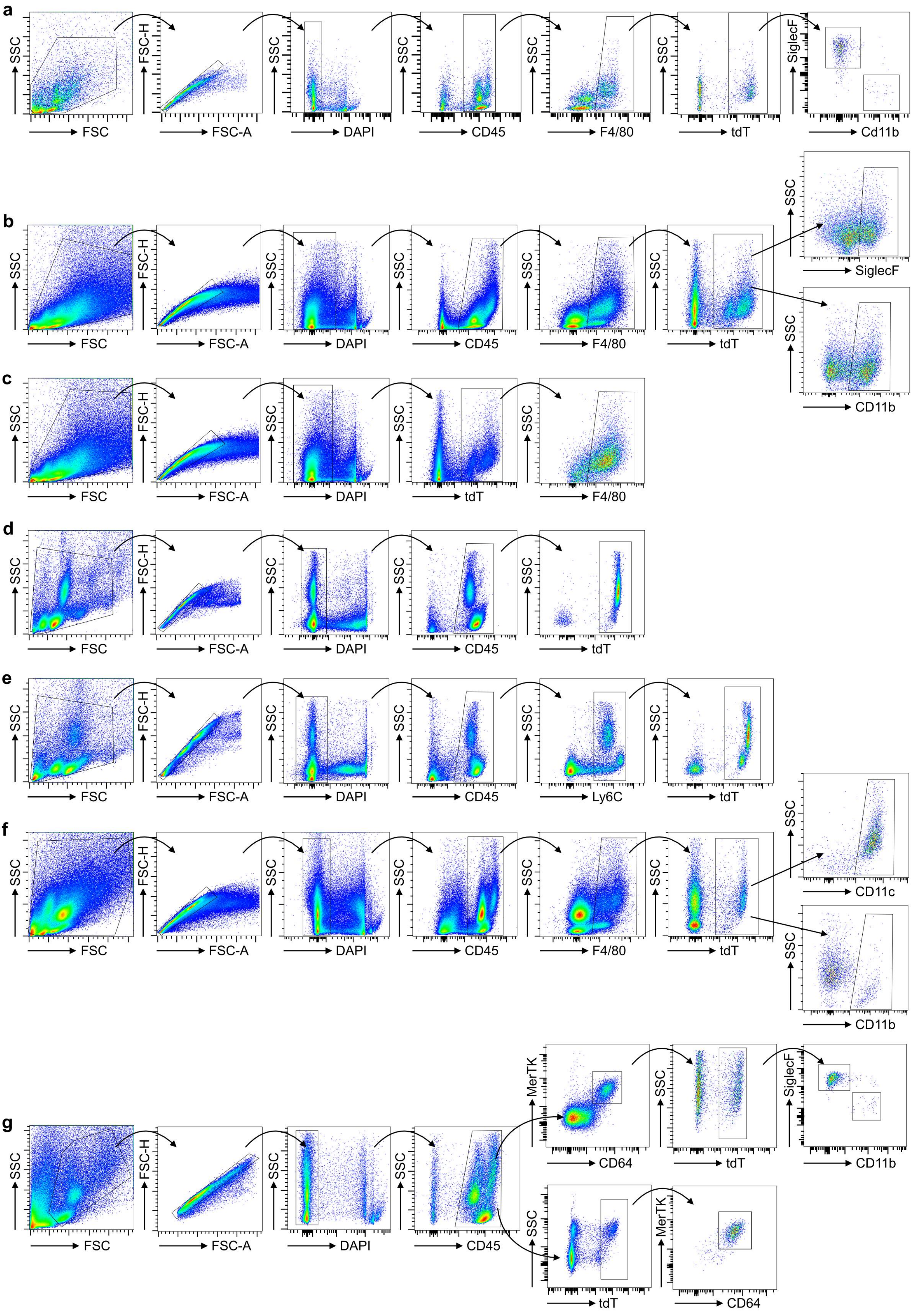
a
SSC
FSC
FSC-H
FSC-A
DAPI
CD45
F4/80
tdT
SiglecF
Cd11b
b
SiglecF
CD11b
c
d
e
Ly6C
f
CD11c
g
MerTK
CD64

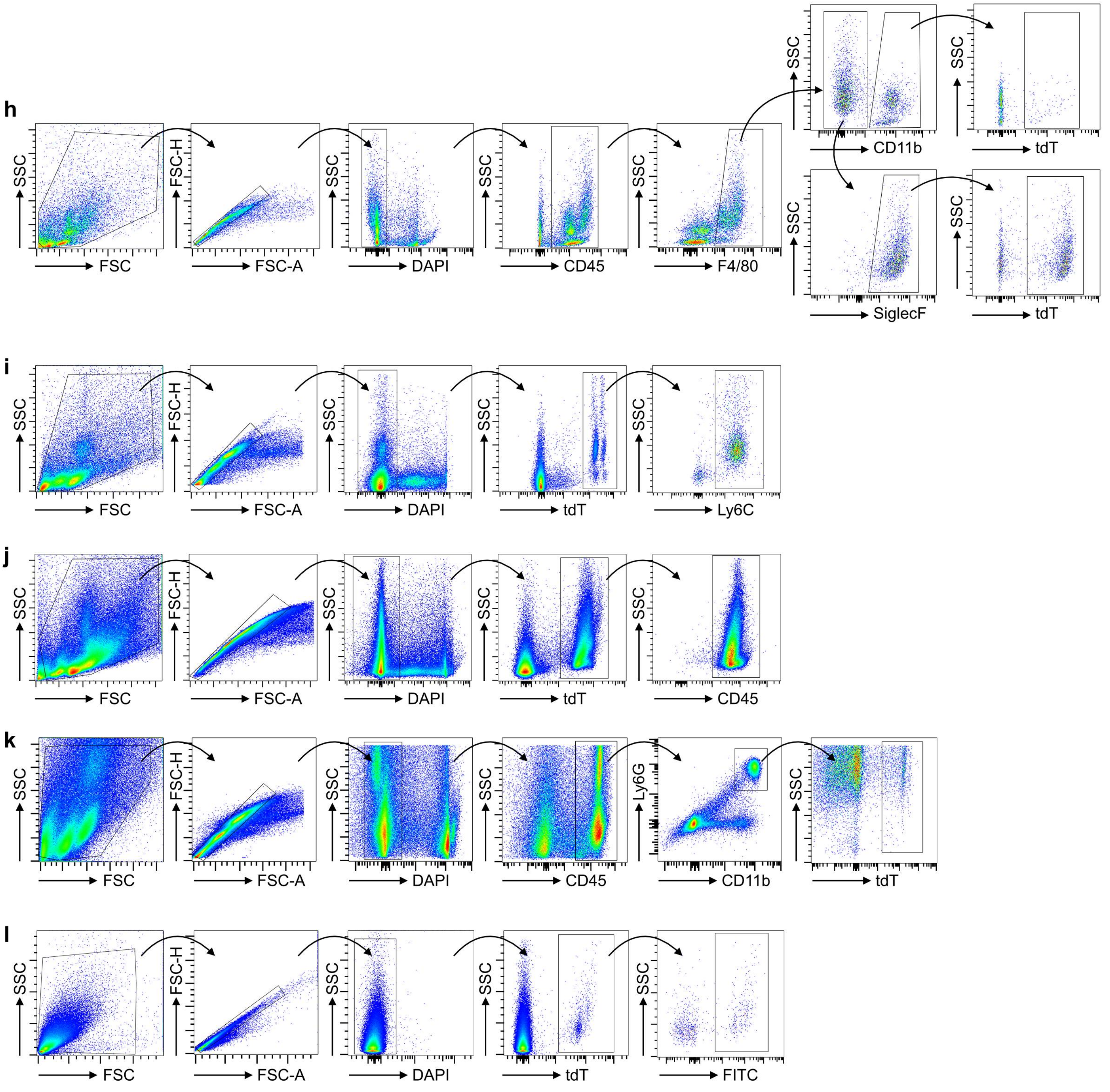

Supplementary Fig. S16. Gating strategies for FACS data. a Gating strategy to analyze the percentages of tdT$^+$, tdT$^+$SiglecF$^+$CD11b$^-$, and tdT$^+$SiglecF$^-$CD11b$^+$ cells in lung macrophages. For Fig. 1d, and Supplementary Fig. S11b for isolating Mo-AMs and Mo-Ims. **b** Gating strategy to analyze the percentages of tdT$^+$, tdT$^+$SiglecF$^+$, and tdT$^+$CD11b$^+$ cells in lung macrophages. For Fig. 2g, h, Fig. 3f, g, Fig. 5g, Fig. 6h, Fig. 7h, Supplementary Fig. S1e (Efficiency), Supplementary Fig. S3d (Lung), Supplementary Fig. S6h (Efficiency), Supplementary Fig. S6i, and Supplementary Fig. S13b. **c** Gating strategy to analyze the percentage of tdT$^+$ cells expressing F4/80. For Fig. 1e, and Supplementary Fig. S1e (Specificity). **d** Gating strategy to analyze the percentage of tdT$^+$ cells in blood CD45$^+$ cells. For Fig. 1f. **e** Gating strategy to analyze the percentage of tdT$^+$ cells in blood monocytes. For Fig. 5c, Fig. 6d, Fig. 7d, Supplementary Fig. S3d (Blood), Supplementary Fig. S6f (Efficiency), and Supplementary Fig. S6g (Efficiency). **f** Gating strategy to analyze the percentage of tdT$^+$, tdT$^+$CD11c$^+$, and tdT$^+$CD11b$^+$ cells in lung macrophages. For Supplementary Fig. S1l. **g** Gating strategy to analyze the percentage of tdT$^+$CD64$^+$MerTK$^+$, tdT$^+$SiglecF$^+$and tdT$^+$CD11b$^+$ cells in lung macrophages. For Supplementary Fig. S2b, c. **h** Gating strategy to analyze the percentage of tdT$^+$ cells within CD45$^+$F4/80$^+$CD11b$^+$ and CD45$^+$F4/80$^+$CD11b$^-$SiglecF$^+$ lung macrophages. For Supplementary Fig. S2d. **i** Gating strategy to analyze the percentage of tdT$^+$ cells expressing Ly6C. For Supplementary Fig. S6f (Specificity). **j** Gating strategy to analyze the percentage of tdT$^+$ cells expressing CD45. For Supplementary Fig. S6h (Specificity). **k** Gating strategy to analyze the percentage of tdT$^+$ cells within CD45$^+$CD11b$^+$Ly6G$^+$ neutrophils. For Supplementary Fig. S10d. **l** Gating strategy to analyze the percentage of FITC$^+$ cells in tdT$^+$ Mo-AMs and Mo-IMs. For Supplementary Fig. S12c, d.

Supplementary Table 1

Primers used for quantitative PCR.

Gene	Forward	Reverse
Gapdh	CCTTCCGTGTTCCTACCCC	GCCCAAGATGCCCTTCAGT
Arg1	CATTGGCTTGCGAGACGTAGAC	GCTGAAGGTCTCTTCCATCACC
Chil3	CCAGCAGAAGCTCTCCAGAAG	CTCCCTTCTATTGGCCTGTCC
Il6	CTGCAAGAGACTTCCATCCAG	AGTGGTATAGACAGGTCTGTTGG
Il1b	GAAATGCCACCTTTTGACAGTG	TGGATGCTCTCATCAGGACAG
Fn1	ATGTGGACCCCTCCTGATAGT	GCCCAGTGATTTCAGCAAAGG
Col3a1	CTGTAACATGGAAACTGGGGAAA	CCATAGCTGAACTGAAAACCACC
Pdgfra	AGGTATGTATCCACACATGCGT	AGTTCCTGTTGGTTTCATCTCG
Pdgfrb	AGCGTGTGGACAGACATGAT	GGTAATCCCGTCAGCATCTT
Rbpj	GAATTTCCACGCCAGTTCAC	ATACAGGGTCGTCTGCATCC
Ctnnb1	CCCAGTCCTTCACGCAAGAG	CATCTAGCGTCTCAGGGAACA
Inos	GCCCCTGGAAGTTTCTCTTC	GTCGATGTCACATGCAGCTT
Tnfa	CTGAACTTCGGGGTGATCGG	GGCTTGTCACTCGAATTTTGAGA
Il10	GCTCTTACTGACTGGCATGAG	CGCAGCTCTAGGAGCATGTG
